# Changes in distinct brain systems identified with fMRI during smoking cessation treatment with varenicline: a review

**DOI:** 10.1007/s00213-024-06556-2

**Published:** 2024-03-02

**Authors:** Vassilis N. Panagopoulos, Alexis Bailey, George K. Kostopoulos, Andreas A. Ioannides

**Affiliations:** 1 Laboratory for Human Brain Dynamics, AAI Scientific Cultural Services Ltd., Nicosia, Cyprus; 2 Department of Physiology, Medical School, University of Patras, Patras, Greece; 3 Pharmacology Section, St. George’s University of London, London, UK

**Keywords:** Varenicline, Functional MRI, fMRI, Nicotine use disorder, Smoking, Smoking cessation

## Abstract

**Background:**

Varenicline is considered one of the most effective treatment options for smoking cessation. Nonetheless, it is only modestly effective. A deeper comprehension of the effects of varenicline by means of the in-depth review of relevant fMRI studies may assist in paving the development of more targeted and effective treatments.

**Methodology:**

A search of PubMed and Google Scholar databases was conducted with the keywords “functional magnetic resonance imaging” or “fMRI”, and “varenicline”. All peer-reviewed articles regarding the assessment of smokers with fMRI while undergoing treatment with varenicline and meeting the predefined criteria were included.

**Results:**

Several studies utilizing different methodologies and targeting different aspects of brain function were identified. During nicotine withdrawal, decreased mesocorticolimbic activity and increased amygdala activity, as well as elevated amygdala-insula and insula-default-mode-network functional connectivity are alleviated by varenicline under specific testing conditions. However, other nicotine withdrawal-induced changes, including the decreased reward responsivity of the ventral striatum, the bilateral dorsal striatum and the anterior cingulate cortex are not influenced by varenicline suggesting a task-dependent divergence in neurocircuitry activation. Under satiety, varenicline treatment is associated with diminished cue-induced activation of the ventral striatum and medial orbitofrontal cortex concomitant with reduced cravings; during the resting state, varenicline induces activation of the lateral orbitofrontal cortex and suppression of the right amygdala.

**Conclusions:**

The current review provides important clues with regard to the neurobiological mechanism of action of varenicline and highlights promising research opportunities regarding the development of more selective and effective treatments and predictive biomarkers for treatment efficacy.

## Introduction

Nicotine use disorder is very frequent, and is clearly associated with significant morbidity and mortality (American Cancer Society 2015). It is estimated that globally exposure to smoking is responsible for approximately 7.1 million deaths per year (National Center for Chronic Disease et al. 2014). Unfortunately, this continues to be the case in spite of widespread implementation of evidence-based public health measures, including public health media campaigns, heavy taxing on tobacco products and extensive restrictions on tobacco use in public areas (Community Preventive Services Task Force 2014, 2015).

In addition, it should be mentioned that nicotine use disorder is a fairly treatment-resistant illness (Prochaska and Benowitz 2016). Approximately more than 70% of smokers would like to quit, but only 40% attempt to do so annually. Unfortunately, approximately only 5% of smokers are ultimately successful in quitting. In spite of the fact that there are available, FDA-approved treatments, most smoking cessation attempts to quit smoking are unassisted with a 12-month abstinence rate of approximately 7% (Zhu et al. 2000). In other words, nicotine addiction is undertreated, and relapse is very common (Prochaska and Benowitz 2016).

The newest available medication for smoking cessation and the focus of the current review is varenicline. Varenicline is a partial agonist of the α4β2 nAchR and α6β2 nAChR receptors as well as a full agonist at α7 nAchRs (Crunelle et al. 2009; Kaur et al. 2009). Clinically, it can decrease nicotine withdrawal symptoms and cravings (Cahill et al. 2016), and can reverse the withdrawal-induced cognitive impairment and negative affect which may in fact contribute to its increased efficacy (Patterson et al. 2009a; Loughead et al. 2010)., The α4β2 nAchR is responsible for the rewarding effect of nicotine via a mechanism involving dopamine release in the reward region of the brain, the nucleus accumbens. As such, α4β2 nAchRs are considered as a major player in the development of nicotine use disorder. Varenicline activates this receptor with a maximal effect of approximately 50% of that of a full agonist, such as nicotine, thus decreasing the withdrawal symptoms as well as the cravings for nicotine. At the same time, it blocks nicotine from exerting its action on the receptor in the case of a lapse or relapse during treatment, thus decreasing the rewarding effect of cigarettes (Cahill et al. 2016). Varenicline appears to be more effective than smoking cessation treatment with other FDA-approved pharmacotherapies including bupropion or a single form of nicotine replacement treatment (NRT) whereas it appears to be comparable in terms of efficacy to combination NRT (Gonzales et al. 2006; Fiore and Jaen 2008; Nides et al. 2008). More specifically, the success rate for abstinence following treatment for 9–24 weeks is approximately 21.8% with varenicline, 16.2% with bupropion, 15.7% with the nicotine patch and 9.4% with placebo (Anthenelli et al. 2016). In spite of reports for infrequent neuropsychiatric side effects associated with varenicline, including suicidal ideation, the medication is generally considered as a safe smoking cessation treatment and is being prescribed even to patients who suffer from psychiatric illness under proper monitoring (Anthenelli et al. 2016; Cahill et al. 2016). In conclusion, varenicline is considered as a first-line treatment option by many experts in the field, alone or in combination with NRT (Hughes 2013). In the current review, head to head comparisons between varenicline and the other two smoking cessation medications will be carried out where appropriate in order to disentangle the unique properties of varenicline. The results of studies on the other two smoking cessation pharmacotherapies will be referenced when relevant in terms of clarifying the results of varenicline.

In the field of addiction research, fMRI is a neuroimaging technique that has been used extensively in part due to its non-interventional nature (Suckling and Nestor 2017). It allows for the assessment of regional brain activity during a particular task or in the resting state following pharmacologic treatment, thus enabling comparisons to placebo controls and to baseline. It thus provides valuable hints in terms of understanding the mechanism of action of a particular intervention and links between neurobiology and behavior (Suckling and Nestor 2017).

An important concept in interpreting treatment effects and mechanisms of action in Biological Psychiatry is the distinction between state-like and trait-like characteristics (Zilcha-Mano et al. 2022). For instance, if a particular brain function pattern is observed in a smoker only during acute nicotine withdrawal, it would be considered a state-like characteristic, as it depends on the state of the smoker. It could be present during the withdrawal state and absent during the satiated state for instance. To add to the complexity of the matter, nicotine fully stimulates the cholinergic nicotine receptor, whereas varenicline does so as a partial agonist, thus the level of nicotine stimulation and satiety can vary as well depending on the pharmacological agent. In contrast, if a particular brain function pattern occurs irrespectively of the state of stimulation of the smoker with nicotine (withdrawal versus satiety) and is absent in non-smokers, then that would be considered a trait-like characteristic. In other words, this would be considered a trait of nicotine use disorder per se, for instance being present in smokers but not in non-smokers. In essence, state-like qualities represent within-individual variance whereas trait-like qualities represent between-individuals variance (Zilcha-Mano et al. 2022). This is an important consideration in the current context as many of the effects of chronic nicotine use, nicotine withdrawal or the particular effects of a specific medication such as varenicline on the brain as depicted with fMRI imaging can be fleeting and dependent upon the particular state of the individual during the imaging session.

The focus of the current review is the presentation and interpretation of findings from a body of fMRI literature concentrating on varenicline effects on brain activity in relation to its therapeutic action. It is an attempt to better comprehend its mechanism of action and elucidate the pathophysiology of nicotine use disorder. Given the grave public health consequences of nicotine use disorder, advancement in the field in terms of developing more effective treatments is urgently needed as has been expressed in prior related reviews (Menossi et al. 2013). An update of this prior work and a comprehensive review of the latest findings of additional functional imaging studies may provide a helpful synopsis for future researchers and for research-oriented clinicians in addressing this unresolved clinical challenge.

## Survey methodology

**Fig. 1:**
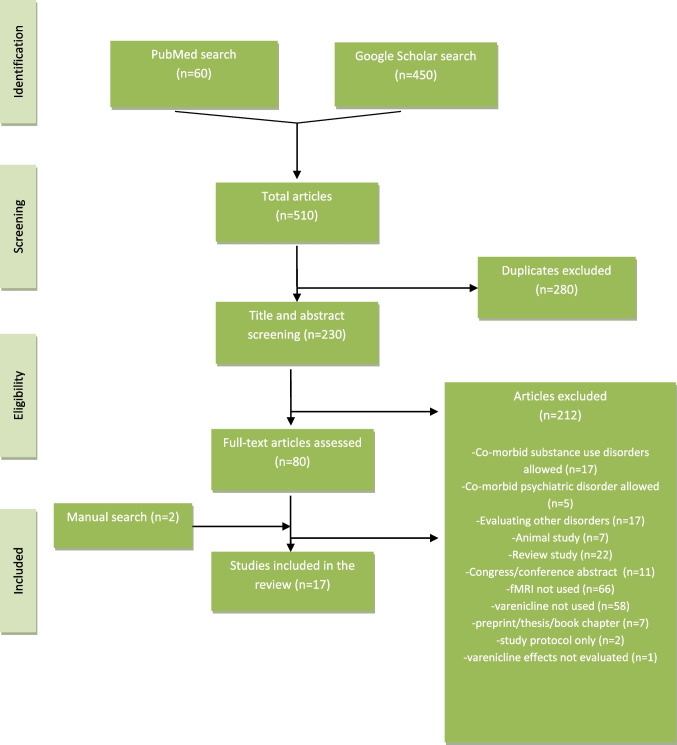
Flow diagram of the screening and selection process

A search of PubMed and Google Scholar databases was conducted with the keywords “functional magnetic resonance imaging” OR “fMRI”, AND “varenicline” to identify any relevant articles in the literature. The most recent search was completed in November of 2023. Only original studies published in the English language regarding the assessment of smoking cessation treatment with varenicline via means of functional magnetic resonance imaging (fMRI) were considered in the current review. Excluded a priori from this review were studies evaluating concurrently co-morbid substance use disorders or major psychiatric disorders other than nicotine use disorder, studies written in languages other than English, other reviews, congress/conference abstracts, animal studies and studies using other imaging methods. The decision to exclude studies with a second co-occurring substance use disorder was made because nicotine use disorder and the related brain function aberrations are already complicated per se. It would be extremely difficult to disentangle the cause of any brain changes in the presence of more than one substance use disorder. The data of individual studies was not combined as there is significant heterogeneity in the study design among different studies that precluded utilization of such methods. All the studies found on PubMed were screened for eligibility. Only the first 200 studies retrieved from Google Scholar in each of the two keyword searches were screened for eligibility after the search results were sorted by relevance for practical reasons. Any references of the selected studies that appeared pertinent were also screened for eligibility and included in the current review if deemed appropriate. The first author read the abstracts of the studies of the aforementioned keyword search results. The first and last author read the full-text articles selected for further eligibility assessment. The predefined inclusion and exclusion criteria were applied and all resulting articles were included in the current review. Figure 1 provides a detailed visual display of the selection process.

## Results

Below follow the results of the current survey organized according to the brain system/process involved. The results for each brain system are also presented in summary in Tables 1, 2, 3, 4, 5, 6, 7, and 8.

**Table 1:** Summary of findings of fMRI Studies in smokers undergoing treatment with varenicline involving the reward system

	Study methodology	fMRI type & task	Main comparison	Sample size & demographics	Sample characteristics	Major findings
Franklin et al. 2011	Double-blind, randomized, placebo-controlled Smoked prior to testing	-CASL perfusion fMRI -Task: audiovisual cues related to smoking and neutral cues -Target function: associative learning/ reward processing	Varenicline VS placebo at rest and after exposure to smoking related video cues, at baseline and after 21 days of treatment	*N* = 22 Average age: 36.1 (2.2) Gender: 73% male, 27% female	Non treatment seeking smokers Average Baseline use: 17.5 (1.6) cigs/day No psychiatric disorders allowed	-Increase in cue-induced cravings correlated with activation of the PCC at baseline -Tx with varenicline associated with increased activation of the ACC, the PCC, the inferior, middle and upper frontal gyri, the lOFC and the dorsolateral PFC with exposure to smoking cues - Tx with varenicline correlated with diminished cue-induced ventral striatum and mOFC activation and cravings - The varenicline-induced activation of the lOFC at rest predicted the diminished response to smoking cues of the mOFC
Hartwell et al. 2013	Open-label, not placebo-controlled Last cigarette 2 h prior to the scan Abstinence verified weekly	BOLD fMRI Smoking, neutral, and rest visual cues shown under the Craving or Resist Condition Target function: Reward processing	Abstinent VS nonabstinent subgroup at week 5 of treatment	*N* = 21 Average age: 35.2 (12.1) Gender: 57% female, 43% male	Treatment seeking smokers Average Baseline use: 20 (2.0) cigs/day “Current, significant” psychiatric disorders not allowed	-During the baseline Resist Condition, the abstinent group showed activation of a distributed network involved in alertness, learning and memory as compared to the nonabstinent subgroup -In the abstinent subgroup, increased activation of the bilateral superior frontal gyrus extending into the PFC was detected at baseline as compared to week 5 under the Resist Condition
Fedota et al. 2015	Double-blind, two-drug, placebo-controlled, cross-over design Last cigarette 12 h prior to scan Nicotine or placebo patch prior to scan on Day ~ 17 of treatment	BOLD fMRI Revised monetary incentive delay task Target function: anticipatory reward-based learning	Smokers VS nonsmokers ± varenicline or nicotine patch or placebo	-24 smokers VS 20 nonsmokers -Average age: Smokers: 36 (10) Vs Nonsmokers: 31 (7) -Gender: Smokers: 52% female, 48% male Nonsmokers: 50% female, 50% male	Non-treatment-seeking smokers Average Baseline use: 16.41 (7.70) cigs/day No psychiatric disorders allowed	-Decreased activation with both positive and negative valence cues in acutely abstinent smokers VS nonsmokers in the left nucleus accumbens, the right putamen, the bilateral ACC -Decreased activation with positive valence cues in the bilateral caudate in acutely abstinent smokers VS nonsmokers smokers -Decreased activation for negative valence cues in the left caudate in acutely abstinent smokers VS nonsmokers -Decreased gain magnitude processing in the ACC in acutely abstinent smokers
Flannery et al. 2019	Double-blind, two-drug, within-subject crossover placebo-controlled Last cigarette ~ 14 h prior to scan Nicotine or placebo patch prior to scan on Day ~ 17 of treatment	BOLD fMRI Performance feedback task Target Brain function:: reward processing	Smokers VS nonsmokers ± varenicline or nicotine patch or placebo	24 smokers VS 20 nonsmokers Average age: Smokers: 36(10) Vs Nonsmokers: 30 (7) Gender: Smokers: 50% female, 50% male Nonsmokers:50% female, 50% male	Non-treatment-seeking smokers Average Baseline use: (18) 8 cigs/day No psychiatric disorders allowed	- Less responsivity of the bilateral ventral striatum to positive feedback in overnight-abstinent smokers than controls and this was not alleviated by varenicline - The aforementioned alteration was correlated with addiction severity -Higher responsivity of the left insula to negative feedback in overnight-abstinent smokers than controls - Increased habenular activity was correlated with cravings for nicotine in the overnight-abstinent smokers group and was alleviated by nicotine
Lesage et al. 2017	Double-blind, placebo-controlled, two-drug, cross-over design Last cigarette ~ 12 h prior to the scan ~ 17 days of Tx with varenicline	BOLD fMRI Probabilistic reversal learning task Target function: Cognitive flexibility, reward sensitivity	Smokers VS nonsmokers ± varenicline or nicotine patch or placebo	24 smokers VS 20 nonsmokers Average age: Smokers: 36(10) Vs Nonsmokers: 30 (7) Gender: Smokers: 50% female, 50% male Nonsmokers:50% female, 50% male	Non-treatment-seeking smokers Average Baseline use: (18) 8 cigs/day No psychiatric disorders allowed	- Smokers showed a bias towards response shifting during acute nicotine withdrawal suggesting increased impulsivity; these aberrations were decreased with varenicline or nicotine - Smokers showed decreased mesocorticolimbic activity before a behavioral shift (ACC, bilateral striatum, anterior insula) associated with cognitive flexibility in comparison to nonsmokers; this was corrected with varenicline or nicotine - Decreased responses to rewards in the bilateral dorsal striatum and ACC indicating decreased reward sensitivity in smokers (VS nonsmokers) were not corrected by nicotine or varenicline. The deficit was associated with severity of addiction

**Table 2:**
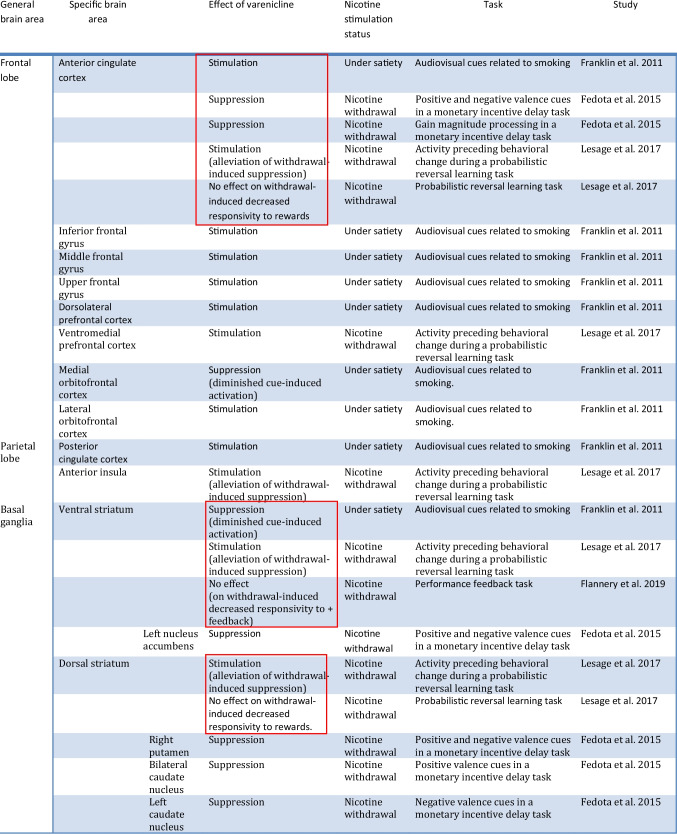
Brain areas affected by varenicline in fMRI studies involving the reward system. Seemingly contradictory findings are highlighted in red. Resting-state data is reported elsewhere

**Table 3:** Summary of findings of fMRI Studies evaluating attention and executive function in smokers undergoing treatment with varenicline

	Study methodology	fMRI type & task	Main comparison	Sample size & demographics	Sample characteristics	Major findings
Lesage et al. 2020	Double-blind, within-subject cross-over randomized order, placebo-controlled 12-h abstinence prior to scanning ~ 17 days of Tx with varenicline	BOLD fMRI Cognitive control tasks Target function: Inhibitory control	Varenicline VS nicotine VS placebo	-Smokers *n* = 24, Gender: 50% female, 50% male Age:36(10) -Nonsmokers: *n* = 20, Gender: 50% female, 50% male, Age: 30 (7)	-Non-treatment seeking smokers -Average Baseline use: 17.7 (7.9) cigs/day -No psychiatric disorders allowed	-Varenicline improved attention deficits only in the Go-Nogo task during acute nicotine withdrawal for lower difficulty tasks -Varenicline did not cause any major changes in fMRI during these tasks
Loughead et al. 2010	-Double-blind, within-subject cross-over randomized order, placebo-controlled -Abstinence verified daily	BOLD fMRI Visual working memory task Target function: Working memory	-Varenicline VS placebo on Day 13 of treatment and following 3 days of mandatory abstinence	*N* = 22 Average age: 41 (13) Gender: 55% male, 45% female	Treatment seeking smokers Average Baseline use: 18.5 (5.3) cigs/day No psychiatric disorders allowed	-Increased activity of the dorsal anterior cingulate, medial frontal and bilateral dorsolateral prefrontal cortices in higher levels of difficulty of a visual working memory task with varenicline during early abstinence - Increased working memory performance was observed with varenicline in highly dependent smokers during early abstinence
Wheelock et al. 2014	Single-arm open label 12-week study Lack of control group	BOLD fMRI & magnetic resonance spectroscopy Stroop color-naming task Target function: Executive function	-Before and after varenicline treatment -Completers VS noncompleters	-11 Completers VS 11 noncompleters -Average age: Completers: 36(11) Vs Noncompleters: 37(13) - Gender: Completers: 64% female, 36% male Noncompleters: 45% female, 55% male	-Treatment-seeking smokers -Average Baseline use: Completers: 23.6 (9.2) cigs/day Noncompleters: 25.4(8) -Psychiatric conditions other than other substance use disorders not excluded	- varenicline Tx associated with decreased fMRI signal in the rostral ACC /mOFC and precuneus/PCC during the Stroop color-naming task - volunteers who dropped out showed increased baseline fMRI BOLD activation in the putamen and insula - varenicline Tx associated with a decrease in dorsal ACC glutamate + glutamine/creatine levels - changes in functional connectivity between the dorsal ACC and regions of the DMN were observed with Tx with varenicline

**Table 4:** Brain areas affected by varenicline in fMRI studies involving attention and executive function

General brain area	Specific brain area		Effect of varenicline	Nicotine stimulation status	Task	Study	
Frontal lobe	Anterior cingulate cortex (ACC)						
		Rostral ACC	Suppression	1 h of abstinence	Inhibitory control/executive function task	Wheelock et al. 2014	
		Dorsal ACC	Stimulation	Under nicotine withdrawal	Visual working memory task	Loughead et al. 2010	
	Medial frontal cortex		Stimulation	Under nicotine withdrawal	Visual working memory task	Loughead et al. 2010	
	Medial orbitofrontal cortex		Suppression	1 h of abstinence	Inhibitory control/executive function task	Wheelock et al. 2014	
	Dorsolateral prefrontal cortex		Stimulation	Under nicotine withdrawal	Visual working memory task	Loughead et al. 2010	
Parietal lobe	Posterior cingulate cortex		Suppression	1 h of abstinence	Inhibitory control/executive function task	Wheelock et al. 2014	
	Precuneus		Suppression	1 h of abstinence	Inhibitory control/executive function task	Wheelock et al. 2014

**Table 5:** Summary of findings of fMRI Studies evaluating emotional processing in smokers undergoing treatment with varenicline

	Study methodology	fMRI type & task	Main comparison	Sample size & demographics	Sample characteristics	Major findings
Loughead et al. 2013	Double-blind, within-subject cross-over randomized order, placebo-controlled Abstinence verified daily	BOLD fMRI Face emotion identification task Target function: emotional regulation	-Varenicline VS placebo on Day 13 of treatment and following 3 days of mandatory abstinence	*N* = 25 Average age: 41 (12.42) Gender: 55% male, 45% female	Treatment seeking smokers Average Baseline use: 18.45 (5.28) cigs/day No psychiatric disorders allowed	- decreased BOLD fMRI signal during a face emotion identification task in the dorsal anterior cingulate cortex, medial frontal cortex, occipital cortex and thalamus, as well as an increase in the middle temporal gyrus with varenicline during early abstinence - reduced amygdala activity with varenicline was observed in a non task-related manner, independent of emotional valence
Sutherland et al. 2013b	Double-blind, placebo-controlled, two-drug, cross-over design Last cigarette ~ 12 h prior to the scan ~ 17 days of Tx with varenicline	BOLD fMRI Emotional face matching paradigm task Target function: Emotional processing of faces	Smokers VS nonsmokers ± varenicline or nicotine patch or placebo	24 smokers VS 20 nonsmokers Average age: Smokers: 36(10) Vs Nonsmokers: 30 (7) Gender: Smokers: 50% female, 50% male Nonsmokers:50% female, 50% male	Non-treatment-seeking smokers Average Baseline use: (18) 8 cigs/day No psychiatric disorders allowed	-Reaction times improved in acutely abstinent smokers with varenicline or nicotine in comparison to nonsmokers -Varenicline did not have any statistically significant effects to the smokers’ group as a whole in terms of amygdala reactivity -Varenicline or nicotine were able to downregulate the abstinence-induced elevated reactivity of the amygdala in the “stable reaction time-improvers” subgroup but did not have an effect in the “variable reaction time improvers” subgroup

**Table 6:**
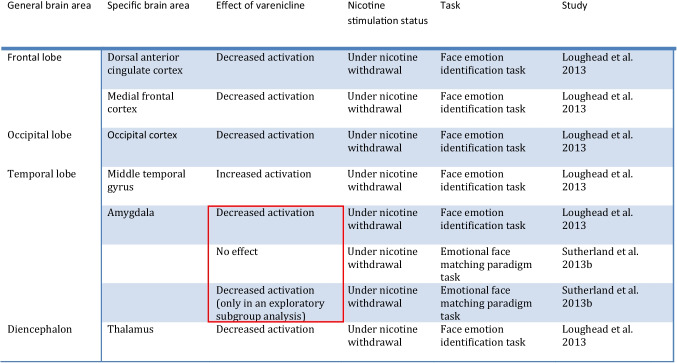
Brain areas affected by varenicline in fMRI studies involving emotional processing. Seemingly contradictory findings are highlighted in red

**Table 7:**
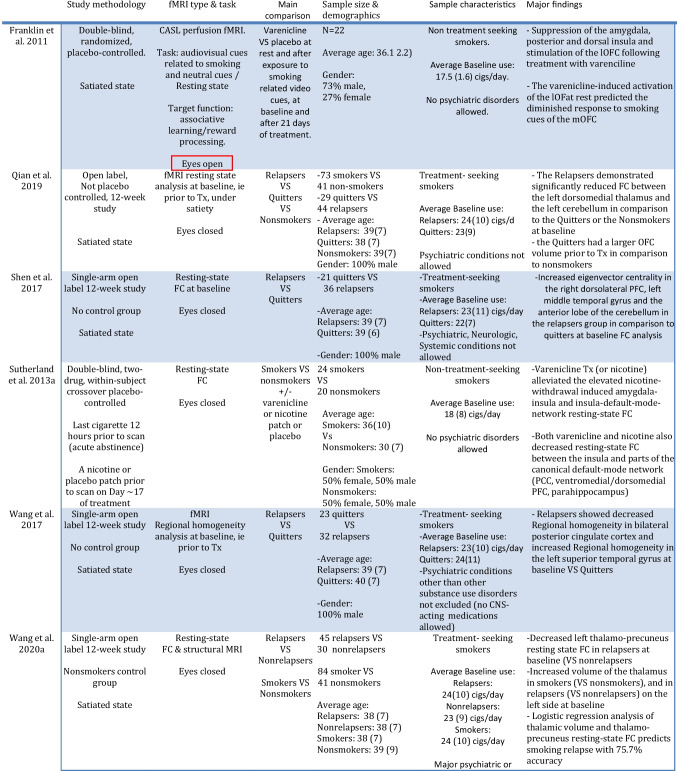

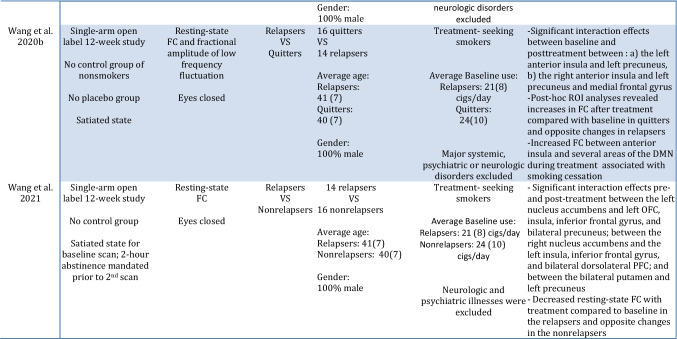
Summary of findings of resting-state fMRI Studies in smokers undergoing treatment with varenicline. Interesting methological difference highlighted in red

**Table 8:**
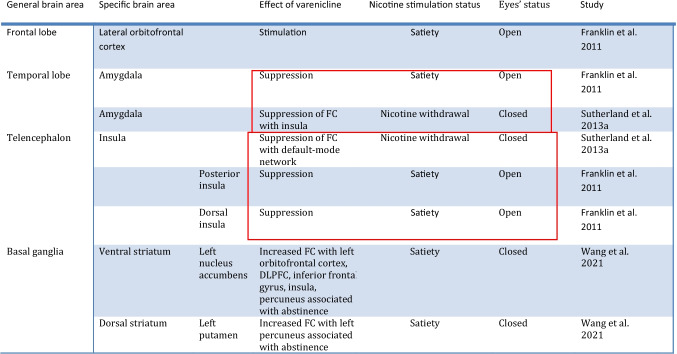
Brain areas affected by varenicline in fMRI studies during the resting state, including functional connectivity changes. Interesting convergent findings in spite of the relevant methodological differences are highlighted in red

### Reward processing

Five fMRI studies were identified focusing on the reward system of the brain. According to the findings of a double-blind, placebo-controlled, crossover study assessing the effects of nicotine and varenicline during a performance feedback task as depicted with BOLD fMRI there is less striatal responsivity to positive feedback in nontreatment–seeking smokers who abstained overnight (*n* = 24) in comparison to nonsmokers (*n* = 20) (Flannery et al. 2019). This change was not alleviated by varenicline (lasting 17 days on average) or nicotine administration in the smokers group and was correlated with addiction severity, hence representing a trait-like aspect of nicotine use disorder. Higher responsivity of the left insula to negative feedback was observed in the smokers group in comparison to controls. Also, nicotine administration (transdermally for 2.9 days on average, with the dose depending on the usual daily nicotine intake) reduced activity in the habenula nucleus both with positive and negative feedback in the smokers group but not in nonsmokers. In addition, increased habenular activity was correlated with the intensity of the cravings and social anhedonia, appearing to be a state-like aspect of the disorder.

In a double-blind, placebo-controlled, cross-over study assessing anticipatory reward processing with BOLD fMRI in non-treatment-seeking smokers receiving treatment with varenicline (*n* = 24), decreased activation was shown in response to both positive and negative valence cues in smokers in comparison to non-smokers in the left nucleus accumbens, the right putamen, and the bilateral anterior cingulate, whereas this effect occurred for positive valence cues in the bilateral caudate, and for negative valence cues in the left caudate (Fedota et al. 2015). Of note, the volunteers were instructed to smoke one last cigarette 12 h prior to the imaging session. In addition, either a nicotine or a placebo transdermal patch was administered prior to the scan on Day 17 to assess the effects of nicotine as well. So, in the absence of the nicotine patch administration, the results pertain to the acute nicotine withdrawal state. Nicotine administration exerted an increased activation of the putamen, did not exert a decrease in anterior cingulate activation in response to reward magnitude, and exerted increased activation of the anterior cingulate in response to anticipated gain vs loss. Based on these results, it appears that varenicline has the ability to decrease the salience of anticipated rewards by decreasing processing of the anterior cingulate cortex (ACC) during nicotine withdrawal, an effect not observed with nicotine suggesting a potentially unique property/mechanism of varenicline which may contribute to its superior efficacy in comparison to nicotine replacement treatment. This of course warrants further investigation. Clinical correlates such as cravings were not included in the current investigation, but it should be mentioned that anterior cingulate activation has been previously reported to correlate positively with cravings in acutely abstinent smokers (McClernon et al. 2009) in accordance with the observed effects in this study and the well-known ability of varenicline to decrease cravings (Cahill et al. 2016).

In a separate double-blind, randomized placebo-controlled trial with 22 non treatment-seeking, smokers, brain function was assessed utilizing CASL perfusion fMRI, following exposure to smoking-related video cues at baseline and following 3 weeks of treatment with varenicline or placebo (Franklin et al. 2011). Of note, smokers who “may be contemplating but who were not currently considering quitting” were recruited. At baseline, both groups exhibited an increase in cravings following exposure to smoking-related cues and this was correlated with an increase in activity of the posterior cingulate cortex (PCC) suggesting involvement of this region in cue-induced cravings, which is consistent with the literature with other drugs of abuse, including cocaine, opioids, alcohol (Garavan et al. 2000; Li et al. 2013; Huang et al. 2018). Treatment with varenicline for 3 weeks was associated with increased activation of the anterior and posterior cingulate cortices, the inferior, middle and upper frontal gyri, the lateral orbitofrontal cortex (lOFC) and the dorsolateral prefrontal cortex (PFC) following exposure to smoking cues relative to nonsmoking-related cues. These activations appear to be varenicline-induced as they were not seen at baseline (i.e. prior to treatment with varenicline) and for the placebo group. The finding of the increased activity of the PCC with varenicline is surprising in light of the known ability of varenicline to decrease cravings (Cahill et al. 2016). At rest, i.e. in the absence of provocation with stimuli but with the eyes open (Personal communication with T Franklin, 2022), increased activation in the bilateral lOFC and decreased activation of the right amygdala and posterior and dorsal insula following treatment with varenicline were observed. In addition, the group receiving varenicline showed diminished activation of the ventral striatum and the medial orbitofrontal cortex (mOFC) and diminished self-reported cravings induced by smoking-related cues in comparison to the placebo group. Cravings were assessed in a subjective manner utilizing the “craving” item of the Schiffman-Jarvik withdrawal scale which was administered before and immediately after exposure to visual cues at baseline (prior to treatment) as well as following varenicline treatment. It should be noted, that the measure used is not a dedicated, validated measure for the evaluation of cravings per se but rather part of a nicotine withdrawal scale (Shiffman and Jarvik 1976). Interestingly, the varenicline-induced activation of the lOFC at rest predicted the diminished response to smoking cues of the mOFC. Of note, in this study the subjects smoked a cigarette of their preferred brand prior to the scan. The researchers intended for the subjects to be non-abstinent to examine the effects of varenicline on cue reactivity in the absence of any nicotine withdrawal related effects, i.e. under the satiated condition (Franklin et al. 2011). Therefore, the results potentially do not provide information as pertinent to fully abstaining smokers during smoking cessation treatment. In addition, basically the combined effects of nicotine and varenicline rather than the effects of varenicline per se are being assessed in this study. Allowing the participants to smoke their own cigarette may provide another source of variability in this study in terms of nicotine content. From the other point of view, varenicline is supposed to be started prior to the planned quit date while smokers are still actively using nicotine (Pfizer Laboratories 2013; Giulietti et al. 2020). Therefore, it is important to assess varenicline’s effects in the presence of nicotine as would be the case in a real-life clinical scenario at the initial stages of treatment. This study underlines the potential involvement of the ventral striatum and the mOFC in the ability of varenicline to reduce cue-induced cravings.

In a study evaluating the effects of varenicline with BOLD fMRI on treatment-seeking smokers undergoing open-label treatment with varenicline (*n* = 16), a task involving the presentation of visual smoking, neutral and rest cues was performed 2 h after their last cigarette (Hartwell et al. 2013). This occurred while the subjects were instructed to either resist the urge to smoke (the Resist Condition) or to allow themselves to have cravings upon presentation of smoking-related visual images (the Crave Condition). During the baseline Resist Condition (i.e. prior to receiving treatment with varenicline), the subgroup of smokers that were able to successfully abstain at 5 weeks (*n* = 10) showed activation of a distributed brain network involved in alertness, learning and memory as compared to the non-abstinent smokers subgroup (*n* = 6). The areas exhibiting this activation included the following: the right insular cortex and possibly the right putamen, the left anterior thalamus, the bilateral middle cingulate, and the posterior cingulate gyrus. In the aforementioned abstinent smokers’ subgroup, increased activation of the bilateral superior frontal gyrus extending into the PFC was detected at baseline as compared to week 5 of treatment under the Resist Condition. Nevertheless, in the absence of a control group, it is impossible to know whether this was an effect induced by abstinence versus varenicline. Also, it is unclear whether the level of nicotine withdrawal (if any) or the effects of nicotine itself, were somehow controlled for prior to each scanning session at baseline for all participants and for the nonabstinent group during the second scan. No statistically significant changes were detected under the Crave Condition when comparing the abstinent and non-abstinent subgroups, but also when comparing baseline and 5-week brain activation patterns in each group individually. This is an unexpected finding in light of the clinical ability of varenicline to reduce cravings and affect reward system-related areas in other fMRI studies (Franklin et al. 2011). Of note, abstinence was confirmed biochemically weekly and at the timepoint of the 5-week scan. The hope is that by running similar baseline testing such as an fMRI under the Resist Condition prior to starting pharmacologic treatment, the outcome could be predicted thus guiding treatment choices based on the probability of response. However, given the limitations of the study including its open-label design, the lack of a control group, the lack of control for the level of nicotine stimulation prior to each session, the relatively small sample sizes while conducting multiple comparisons (before VS after treatment, abstinent VS non-abstinent, under the Resist VS Craving Condition), and the assignment of subjects who dropped out to the non-abstinent group, it is impossible to reach any conclusions that could be generalized to the target patient population. These limitations may also explain the inability to detect statistically significant changes under the Craving Condition.

In a separate randomized, double-blind, placebo-controlled, cross-over study the effects of varenicline and nicotine in regard to reward sensitivity and cognitive flexibility were assessed (Lesage et al. 2017). The treatment with varenicline or placebo lasted for approximately 17 days. The smokers group showed a bias towards response shifting during acute nicotine withdrawal suggesting increased impulsivity. This aberration was decreased with treatment with varenicline or nicotine. In addition, smokers showed decreased mesocorticolimbic activity before a behavioral shift (ACC, bilateral striatum, anterior insula) associated with cognitive flexibility in comparison to nonsmokers, and these changes were corrected with varenicline or nicotine, i.e. this represents a state-like characteristic. During performance of a different task, decreased responses to rewards in the bilateral dorsal striatum and ACC were observed indicating decreased reward sensitivity in smokers during nicotine withdrawal (VS nonsmokers), in line with literature of other drugs of abuse (Volkow et al. 2010). Surprisingly, this deficit was not corrected by nicotine or varenicline and was associated with severity of addiction, ie represents a trait-like facet of nicotine use disorder (Lesage et al. 2017). Based on this evidence, it appears that current pharmacotherapies, namely nicotine and varenicline, are able to mitigate certain deficits observed in reward-based learning but not others.

In summary, it appears that varenicline influences the activity of several key areas involved in reward processing. Namely, following varenicline treatment, when under the satiated state and in response to smoking-related visual cues (vs neutral cues), certain areas are stimulated (the ACC, the PCC, the inferior, middle and upper frontal gyri, the lOFC, the dorsolateral PFC) whereas others exhibit diminished activation (the ventral striatum and the mOFC). The aforementioned diminished activation to smoking-related visual cues of these two important reward circuitry areas was correlated with a reduction in cue-induced cravings scores thus implicating this region-specific activation as a potential mechanism by which varenicline exhibits its anti-craving effect. Also the varenicline-induced increased activation of the lOFC during the resting state correlated inversely with the diminished cue-induced activation of the mOFC. These findings highlight potential neurological underpinnings of varenicline’s mechanism of action (Franklin et al. 2011). Areas involved in alertness, learning and memory may serve as predictors of successful treatment with varenicline (Hartwell et al. 2013). Interestingly, it appears that varenicline does not adequately reverse the reduced responsivity to rewards of the ventral striatum (Flannery et al. 2019), the bilateral dorsal striatum and the ACC (Lesage et al. 2017) which is induced by acute abstinence suggesting that varenicline’s mechanism of action in suppressing nicotine withdrawal symptoms may not involve normalization of the suppressed ventral striatal responsivity evident in abstinent smokers. This may partly explain the limitations of this treatment option for smoking cessation. Whether treatment options focusing at reversing the decreased withdrawal-induced ventral striatal responsivity are more effective warrants further investigation. Interestingly, seemingly contradictory findings are reported with respect to varenicline’s effects on the ventral striatum as they seem to depend on the nicotine exposure status of the protocol (e.g. withdrawal vs. satiety) and the experimental task (please see highlighted items in Table 2). For instance, in one study varenicline appears to suppress the activation of the ventral striatum under satiety in response to smoking cues in comparison to neutral ones (Franklin et al. 2011). In contrast, in a separate study the ventral striatum is stimulated by varenicline during a task evaluating cognitive flexibility under nicotine withdrawal (alleviation of withdrawal-induced suppression) (Lesage et al. 2017). In turn, in a third study, varenicline appears to have no effect on the withdrawal-induced decreased responsivity of this area to positive feedback (Flannery et al. 2019). Hence, it is possible that varenicline can suppress the activity of the ventral striatum only when it is stimulated by smoking cues and not otherwise (for instance when not activated or when suppressed during cognitive flexibility or reward sensitivity testing). Pharmacologically speaking, this may reflect the partial α4β2 nAchR agonist properties of varenicline (Crunelle et al. 2009; Kaur et al. 2009), with varenicline acting as a full agonist to α4β2 nAchRs when nicotine in not present (i.e. during withdrawal) and as an antagonist to nicotine as they compete with each other for the occupancy of α4β2 nAchR under satiety (Coe et al. 2005). In addition, it should be noted that differing results were found in terms of the effect of varenicline to the ACC with one study reporting cue-induced stimulation under satiety (Franklin et al. 2011), whereas others reporting either suppression or stimulation under nicotine withdrawal with different tasks (Fedota et al. 2015; Lesage et al. 2017). Also, in one of these studies, stimulation or no effect was reported on the dorsal striatum during nicotine withdrawal depending on the task being evaluated (Lesage et al. 2017). These discrepancies again likely reflect the difference in nicotine stimulation status of these protocols, the unique partial agonist nature of varenicline and/or the variability of tasks upon which varenicline may exhibit differential effects.

### Working memory, attention & executive function

Three studies probing cognitive aspects were identified and are discussed next. The first study in this set, examined working memory and attention changes, in a cohort of 22 treatment-seeking smokers. This study utilized a within-subject, cross-over, double-blind placebo-controlled design with the visual N-back working memory task. Active treatment was associated with increased BOLD signal in the dorsal ACC, medial frontal and bilateral dorsolateral prefrontal cortices in higher levels of difficulty of the task in hand on Day 13 of treatment as compared with placebo (Loughead et al. 2010). Testing here as well followed a mandatory and verified 3-day period of abstinence from smoking. Varenicline also improved working memory performance during this period of early abstinence in highly dependent smokers but not in less dependent smokers (Loughead et al. 2010) pointing towards a stimulatory effect of ACC as a potential mechanism underlining the memory improvement effect of varenicline in dependent smokers which invariably would suffer from impairment in that domain during abstinence. Pharmacologically speaking, this may reflect the agonist property of varenicline on α7 nAChRs which are highly localized in the ACC, as indicated in a recent preclinical study demonstrating a key role of α7 nAChRs mediating the well-characterized memory-enhancing properties of varenicline (Esaki et al. 2023).

In a relatively small (*n* = 11), open-label smoking cessation study with treatment-seeking volunteers, treatment with varenicline for 12 weeks was associated with decreased levels of glutamate in the dorsal ACC as detected by single-voxel proton magnetic resonance spectroscopy (Wheelock et al. 2014). In addition, treatment with varenicline was associated with decreased activation in the rostral ACC /mOFC and precuneus/PCC during the Stroop color-naming task in comparison to baseline, which is the classic task for testing inhibitory control and executive function (Pardo et al. 1990). Considering the well-documented role of OFC activation in driving automatic drug- taking behavior due to the weakening of top down inhibitory control from executive centers in the PFC (Goldstein and Volkow 2011), the suppression of OFC activation by varenicline may have important implications in suppressing this automatic behavior. Nonetheless, task performance remained unchanged with treatment and as such this hypothesis warrants further investigation. A psychophysiological analysis during task performance with the dorsal ACC serving as seed revealed changes in connectivity between the seed area and regions of the default-mode network. Interestingly, volunteers who did not complete the study showed increased baseline fMRI BOLD activation in the putamen and insula in comparison to those who completed the study providing hints regarding potential biomarkers predictive of treatment failure (Wheelock et al. 2014) which is important especially in light of the fact that the putamen has been implicated in habit formation (Tricomi et al. 2009). This observation is in line with the well-established evidence that the switch from controlled to compulsive drug-taking which is notoriously difficult to treat, represents a transfer of control of drug-taking behaviors from the PFC and the nucleus accumbens to the caudate nucleus and the putamen, in essence representing the establishment of habitual and automatic behaviors (Everitt et al. 2008). Limitations of the Wheelock et al. 2014 study include the small sample size, the single-arm and open-label design, and the lack of a placebo group. In the absence of the latter, it is difficult to know whether the changes observed were induced by abstinence or treatment with varenicline especially taking into account that the majority of the volunteers quit smoking successfully by the end of the study.

In a double-blind, placebo-controlled, two-drug, cross-over study evaluating attention and inhibitory control via BOLD fMRI, in non-treatment seeking smokers and controls (Lesage et al. 2020), varenicline improved attentional deficits only in the Go-Nogo task during acute nicotine withdrawal especially at a lower level of difficulty (a state-like characteristic). When a higher level of inhibitory control was needed, no deficits were seen. No major changes were observed with varenicline in terms of fMRI findings which makes it difficult to associate the attention enhancing effect of varenicline in abstinent smokers with any regional changes in brain activity. Of note, behavioral changes occurred during the less demanding control condition but the task in hand was not designed to test for that.

In summary, active treatment with varenicline appears to improve the cognitive deficits associated with abstinence (Loughead et al. 2010). During cognitive testing, in the context of abstinence, varenicline appears to stimulate the dorsal ACC, the medial frontal and the bilateral dorsolateral PFC (Loughead et al. 2010). Also it appears to decrease the activation of the rostral ACC /mOFC and precuneus/PCC in the absence of nicotine withdrawal (Wheelock et al. 2014). As such, based on the evidence summarized above and the current knowledge of the neurobiology of drug addiction, one is tempted to hypothesize that varenicline may assist smoking cessation by suppressing cognitive deficits via a mechanism which involves restoration of executive centers of the brain (ACC), and as such, restoration of control over reward saliency regions of the brain (OFC). These clues may be especially important with respect to designing and predicting more efficient and targeted approaches for smoking cessation in the future.

### Emotional processing

Two studies were identified focusing on emotional processing using facial expressions of emotion. In the first, the effects of treatment with varenicline were assessed evaluating 25 treatment-seeking smokers in a within-subject, cross-over, double-blind, placebo-controlled design. The main comparison was made on Day 13 between treatment with varenicline and placebo (Loughead et al. 2013). Active treatment was associated with a decreased activation during a face emotion identification task in the dorsal ACC, medial frontal cortex, occipital cortex and thalamus, as well as increased activation in the middle temporal gyrus. Of note, testing followed a mandatory and verified 3-day period of abstinence from smoking. Varenicline treatment was also associated with improved performance in the aforementioned task in terms of correct response time but not performance accuracy. Also, in an exploratory region of interest analysis of the data, it was found that amygdala activation was dampened with varenicline treatment and interestingly no increased activity was demonstrated even with threat-related facial expressions in contrast to what had been expected by the researchers. Also treatment did not appear to have any major effects on the positive and negative mood measures used in the study (Loughead et al. 2013).

In a separate randomized, double-blind, placebo-controlled, cross-over study the effects of varenicline and nicotine in regard to amygdala reactivity were assessed with an emotional face matching paradigm (Sutherland et al. 2013b). The treatment with varenicline or placebo lasted for approximately 17 days. Reaction times improved in acutely abstinent smokers with varenicline or nicotine in comparison to nonsmokers. Varenicline did not have any statistically significant effects to the smokers’ group as a whole in terms of amygdala reactivity. However, after subdividing the groups based on their propensity to show stable versus variable improvement in reaction times, it was shown that varenicline or nicotine was able to downregulate the abstinence-induced elevated reactivity of the amygdala in the stable reaction time-improver’s subgroup. In contrast, they did not have an effect on amygdala reactivity in the variable reaction time improvers’ subgroup. This suggests that some of the effects of varenicline may not be uniform among all smokers but rather that they may be influenced by certain variables. A better understanding of these underlying variables may allow for a more individualized selection of medication based on predictive biomarker testing.

In summary, treatment with varenicline appears to induce decreased activation of the dorsal ACC, the medial frontal cortex, the occipital cortex, the thalamus, and the amygdala during emotional processing tasks in the context of acute abstinence whereas it increases activity of the middle temporal gyrus (Loughead et al. 2013). However, no effect on the amygdala was found in a separate study (prior to implementing a subgroup analysis) (Sutherland et al. 2013b). In other words, these two studies appear to have produced partly inconsistent results with the former study indicating suppression of the nicotine withdrawal-induced activation of the amygdala with varenicline, whereas the latter one finding no such effect (please see highlighted areas in Table 6). Methodological differences including the different duration of abstinence prior to scanning (3 days of abstinence with a strict verification protocol versus 12 h of abstinence respectively), the different functional tasks used and sample differences (treatment VS non-treatment seeking respectively) could potentially account for the differences in the findings. Given that nicotine withdrawal is characterized by negative affect and heightened emotional reactivity which increase the possibility of a relapse, these studies provide interesting hints in terms of the ability of varenicline to improve emotional regulation (Lerman and Audrain-McGovern 2010). Further studies are warranted to identify the effects of varenicline on amygdala activity, which is well known to be involved in negative emotional states, craving and thw negative reinforcement stages of drug addiction (Koob and Volkow 2016).

### Resting state

Finally, seven studies are reviewed, all relying on fMRI resting-state functional connectivity (FC) measures to evaluate treatment with varenicline. The first of these studies utilized a double-blind, placebo-controlled design of nontreatment-seeking smokers following overnight abstinence (Sutherland et al. 2013a). Nicotine withdrawal was correlated with increased functional amygdala-insula and insula-default-mode network interactions. This is important especially in the context of the well-known associations of FC aberrations of the amygdala with inadequate sleep and insomnia, internalizing symptoms and depression (Paulus and Stein 2010; Huang et al. 2012; Klumpp et al. 2018), which also represent key physical and emotional symptoms of nicotine withdrawal. Treatment with varenicline (or nicotine) decreased amygdala-insula resting-state FC as well as resting-state FC between the insula and constituents of the canonical default-mode network (PCC, ventromedial/dorsomedial PFC, parahippocampus). Thus, nicotine withdrawal appears to be inducing changes amenable to smoking cessation treatment with NRT or varenicline, thus representing state-like characteristics. It is hypothesized that the reported FC effect of varenicline is associated with suppression of relevant withdrawal symptoms. As such, these observations have implications for the design of future effective anti-withdrawal therapy by targeting the weakening of the amygdala-insula resting-state FC.

In an open-label 12-week study, evaluating the effects of smoking cessation treatment with varenicline in 55 exclusively male, treatment-seeking smokers, it was shown that at baseline (i.e. prior to receiving treatment with varenicline) the smokers who did not successfully quit by week 12 demonstrated decreased Regional homogeneity in the bilateral PCC and increased Regional homogeneity in the left superior temporal gyrus in comparison to the smokers who successfully quit by week 12 (Wang et al. 2017). Smoking was allowed ad libitum prior to scanning. These results provide clues regarding potential biomarkers for predicting treatment efficacy in a personalized approach. Limitations include single-arm, open-label design with exclusively male participants. In the absence of a placebo control group, it is unclear whether these markers relate to relapse vulnerability in general irrespectively of varenicline treatment versus treatment efficacy specifically for varenicline.

In a separate, open-label 12-week study, assessing the effects of varenicline in treatment-seeking male smokers (Wang et al. 2021), changes in resting-state FC were assessed. Smoking was allowed ad libitum for the baseline scan whereas smoking was not allowed for 2 h prior to 2nd scan. Significant interaction effects between pretreatment baseline and posttreatment were detected between: a) the left nucleus accumbens and left orbitofrontal cortex (OFC), insula, inferior frontal gyrus, and bilateral precuneus b) the right nucleus accumbens and the left insula, inferior frontal gyrus, and bilateral dorsolateral PFC, c) the bilateral putamen and left precuneus. Depending on treatment outcome (continuous abstinence during the last 4 weeks of treatment), the group was divided to relapsers and nonrelapsers. In the relapsers’ subgroup, decreased resting-state FC between the nucleus accumbens and the OFC, the dorsolateral PFC, the IFG, the insula and the precuneus was shown following treatment compared to baseline. In contrast, the nonrelapsers subgroup showed opposite longitudinal changes. These results indicate that increased striatal resting-state FC with the aforementioned areas following treatment may be associated with improved varenicline treatment outcomes. In light of the fact that nicotine is known to stimulate activity in the nucleus accumbens, the OFC, the dorsolateral PFC and the insula (Brody et al. 2002; Hayashi et al. 2013), one would expect that in the absence of nicotine and the presence of the partial agonist, varenicline, the nonrelapsers group would show decreased FC between these areas following treatment, but this was not the case. At the same time, the effects of varenicline following treatment as explained previously appear to be highly dependent upon the nicotine stimulation state and the task being performed including the stimulation with cues, whereas the current study was completed under very different conditions (resting state, variable degree of abstinence). It should be noted also that the interpretation of the results is further complicated by the fact that it is unclear whether the differences observed in the 2 groups are related to treatment with varenicline per se, the effects of nicotine or smoking cessation in the nonrelapsers group. In addition, in the context of the strict criterion of 4 weeks of abstinence for assignment to the nonrelapsers group, it should be noted that some of the relapsers could potentially have been abstaining for up to almost 4 weeks from nicotine at the time of the second scanning session. As such, the degree of abstinence and the effects of nicotine could have been very variable in this subgroup.

In a 12-week, open-label smoking cessation treatment study with varenicline with 41 nonsmokers and 84 treatment-seeking male smokers, increased volume of the thalamus was detected in smokers in comparison to nonsmokers, and in relapsers in comparison to nonrelapsers on the left side. Relapse was similarly defined as non-abstinence during the last 4 weeks of treatment. Smoking was allowed ad libitum prior to scanning. Also, decreased left thalamo-precuneus resting state FC was detected in relapsers when compared to nonrelapsers at baseline, i.e. prior to varenicline treatment. Interestingly, logistic regression analysis of the thalamic volume and thalamo-precuneus resting state FC was able to predict smoking relapse with 75.7% accuracy. Together, these findings point towards high thalamic volume and decreased thalamo-precuneus resting state FC as a potential predictive biomarker for treatment resistance and relapse. Future studies should focus on assessing the efficacy of targeted approaches focusing on reversing these changes in the hope of improving treatment outcomes. Limitations include single-arm, open-label design and including exclusively Han Chinese, male, right-handed participants (Wang et al. 2020a). Similarly to earlier, in the absence of a placebo group, it is unclear whether the results pertain to relapse vulnerability in general versus the treatment efficacy of varenicline per se. Also, given the specific demographic characteristics of the sample, it is unclear whether these results would apply to the general smoking population.

In a separate, open-label 12-week study, assessing the effects of varenicline in 30 treatment-seeking male smokers (Wang et al. 2020b), changes in resting-state FC were assessed. Significant interaction effects comparing pretreatment baseline and posttreatment were observed a) between the left anterior insula and left precuneus and b) between the right anterior insula and left precuneus and medial frontal gyrus. Post-hoc region-of-interest analyses in the aforementioned brain areas showing interaction effects revealed increases in FC after treatment compared with baseline in quitters and opposite longitudinal changes in relapsers. Increased connectivity between the anterior insula and several areas of the default mode network during treatment was associated with improved prognosis in terms of smoking cessation again pointing towards assessing the impact of targeting the strengthening of these FCs in the future, as a potential therapeutic approach to smoking. Limitations include single-arm, open-label design and including exclusively male participants which render the results preliminary as explained earlier in terms of generalizibility.

In a 12-week smoking cessation treatment study with varenicline, the subgroup of smokers that were not able to remain abstinent for the last 4 weeks of treatment, demonstrated significantly reduced FC between the left dorsomedial thalamus and the left cerebellum in comparison to the quitters’ group as illustrated via resting state fMRI at baseline (prior to treatment) and under satiety (Qian et al. 2019). In the quitters’ group the FC of the aforementioned areas was somewhat lower than that of the nonsmokers’ group.Limitations include single-arm, open-label design and including exclusively male participants.

In another 12-week smoking cessation study with varenicline, a FC of the baseline fMRI scans of 57 treatment-seeking smokers (prior to a quit attempt) was performed comparing the smokers who successfully quit by 12 weeks (quitters) and the ones who did not (relapsers) (Shen et al. 2017). Smoking was allowed ad libitum prior to scanning. Increased eigenvector centrality was identified in the right dorsolateral PFC, left middle temporal gyrus and the anterior lobe of the cerebellum in the relapsers group in comparison to quitters. Limitations include single-arm, open-label design and including exclusively male participants.

In summary, treatment with varenicline (or nicotine) appears to dampen the increased amygdalae-insula resting-state FC observed during acute abstinence which is likely to be correlated with suppressing increased levels of withdrawal-related anxiety and irritability (Sutherland et al. 2013a). This suggests that treatment options focusing on reducing this connectivity may improve nicotine withdrawal-related symptoms and improve treatment outcomes in relation to smoking cessation. Concurrently, at rest varenicline appears to cause suppression of the activity of the amygdala during the satiated state as well (Franklin et al. 2011). Varenicline also appears to induce suppression of the activity of the insula and its FC with the default-mode network under both withdrawal and satiety during the resting state (Franklin et al. 2011; Sutherland et al. 2013a). These convergent effects on the amygdala and the insula are also highlighted in Table 8 which summarizes the resting state results organized by region. These convergent effects appear to be occurring in spite of the divergent methodology in terms of the eye condition as well. Namely, in all of the included resting-state studies the volunteers kept their eyes closed during imaging except for the Franklin et al. study in which they were instructed to keep them open. Interestingly, the aforementioned connectivity data involving several brain areas including the PCC, the left superior temporal gyrus (Wang et al. 2017), the striatum (Wang et al. 2021), the anterior insula (Wang et al. 2020b), the left thalamo-precuneus FC (Wang et al. 2020a) as well as the FC between the dorsomedial thalamus and the left cerebellum (Qian et al. 2019), suggests that functional imaging may have the potential to predict treatment outcome. This data is presented in concise form in Table 9 and will be addressed further in the Discussion. In terms of limitations, in light of the lack of a placebo group, it is unclear whether the results of several of the aforementioned studies (Shen et al. 2017; Wang et al. 2017, 2020a, 2021; Qian et al. 2019) pertain to relapse vulnerability in general versus the treatment efficacy of varenicline per se. Also, it is unclear whether the differences observed in these studies pertain to the presence/absence of nicotine versus the effect of varenicline, since obviously abstainers and relapsers differed in terms of that variable as well.

**Table 9:**
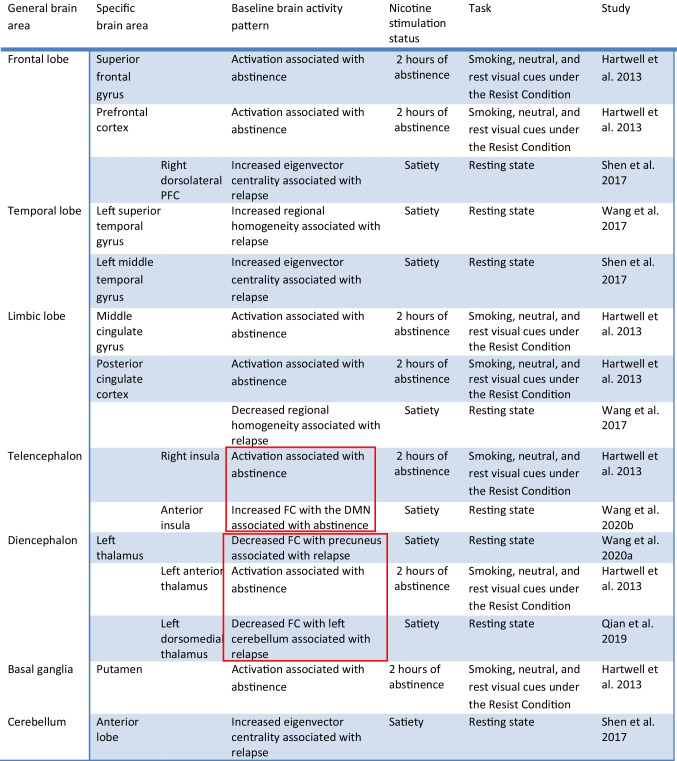
Brain activity patterns at baseline (prior to receiving treatment) including functional connectivity (FC) changes associated with treatment outcome with varenicline in fMRI studies

## Conclusions

As discussed above, nicotine use disorder disrupts the function of multiple brain systems affecting executive function, working memory, affective regulation and reward processing during acute or long term abstinence (Lyvers et al. 2014; Oliver et al. 2017; Martín Ríos et al. 2021) which all play a key role in craving induction and in the motivational trigger to relapse. From a clinical perspective, varenicline has several properties that are thought to contribute to its superior efficacy in smoking cessation addressing many of these aberrations. Namely, it has been shown clinically to reduce cravings for nicotine, decrease nicotine withdrawal symptoms, including negative affect, working memory and attention deficits but also to decrease the level of satisfaction should a lapse occur (Oncken et al. 2006; Patterson et al. 2009a). Evidence from functional imaging further reinforces these clinical observations and begins to unravel their neurophysiologic foundations. The current review presents the main findings of the studies, pointing out apparent inconsistencies which represent areas where more research is needed.

### Impact of varenicline on reward processing

It has long been recognized that there are reward processing aberrations in addiction. Positive reinforcement is profoundly derailed, in the form of a decreased ability to enjoy natural rewards and a preference for artificial rewards, e.g. the substance of abuse (Koob and Volkow 2010). At the same time, negative reinforcement is also affected, as manifested by the activation of stress systems leading to negative affective states, including depressed mood, anxiety, irritability, cravings and anhedonia during the withdrawal phase (Goldstein and Volkow 2002; Wise and Koob 2014; Batalla et al. 2017). Based on the results of the current review, during acute abstinence reduced ventral striatal responsivity to positive feedback was observed and this was not alleviated by varenicline (or nicotine) (Flannery et al. 2019). Under the satiated state and in the absence of stimulation, decreased ventral striatum activation was illustrated by the Franklin et al. study following treatment with varenicline (Franklin et al. 2011). This provides once again converging evidence that implicates this area in the pathophysiology of the disorder in different phases of the addictive process, e.g. under nicotine withdrawal or satiety, in the absence or the presence of treatment with varenicline respectively. Interestingly, another pharmacologic agent approved for smoking cessation, bupropion, has also been shown to decrease the cigarette-related cue-induced activation of the ventral striatum under satiety (Culbertson et al. 2011) while unsuccessfully treated smokers on bupropion have been shown to have increased smoking-related cue-induced activity in the ventral striatum (Weinstein et al. 2010). In comparison to the related NRT literature, increased cue-induced activity under satiety has been reported in the ventral striatum at baseline (e.g. prior to treatment) in smokers who ended up achieving abstinence with treatment, and these differences were reversed following treatment (McClernon et al. 2007). The Franklin et al. study is the closest one methodologically that can be compared to these results. It also reported increased activity of the ventral striatum (as well as the PCC) under similar conditions (prior to treatment, with visual cues and under the satiated condition) and following varenicline treatment, this activation of the ventral striatum was diminished also (Franklin et al. 2011). Following NRT, increased activity has also been reported in other areas including the caudate nucleus, the PFC, the primary somatosensory cortex, the temporal cortex, the parietal cortex, and the anterior and PCC (Janes et al. 2009) and diminished stimulation has been observed in the amygdala with smoking-related visual cues (McClernon et al. 2007). According to the Franklin et al. study activations were also reported in common in the dorsolateral PFC, the anterior and posterior cingulate following varenicline treatment; decreased activity of the amygdala was noted in agreement with the previous results (Franklin et al. 2011). These consistent results may point towards common underlying mechanisms of actions of these different pharmacotherapies.

In terms of the function of the ventral striatum, it has been known that dopamine release in this anatomic structure appears to be centrally involved in the reinforcing properties of nicotine and thus the processing of the euphoric response to nicotine (Brody et al. 2004; David et al. 2005). It is also considered to play an important role in the signaling of smoking-related stimuli of the environment (David et al. 2005). The results of our review with varenicline (and bupropion) (Culbertson et al. 2011; Franklin et al. 2011) producing decreased activation of this area under the satiated condition and with the failure of varenicline to reverse the withdrawal-induced effect (Flannery et al. 2019) appear to further highlight the complexity of the matter due to the interplay of multiple variables, including the different levels of nicotine stimulation states (acute withdrawal, satiety), the direct medication effects on brain function (for instance nicotine, varenicline or bupropion), but also the different task used during the fMRI scan in each study. Based on the aforementioned results and its known biological functions that relate to addiction, the ventral striatum continues to represent a very promising target for future studies and for the development of future therapeutics.

In addition, in one of the presented studies, varenicline was shown to reduce the salience of anticipated rewards in contrast to nicotine during acute nicotine withdrawal by decreasing the processing of gain magnitude cues and reward-related processing (Fedota et al. 2015). In other words, it appears that incentive salience, one of the “wanting modules” of the brain (Berridge 2017), is downregulated by varenicline in line with its therapeutic effects on the disorder. This appears to be mediated by a decrease in the activity of the ACC (Fedota et al. 2015). The ACC is involved in attention as it relates to rewarding stimuli and executive control (Hickey and van Zoest 2012). It has also been implicated in the impulsivity associated with substance use disorders (Kaufman et al. 2003; Luijten et al. 2011). Both varenicline and nicotine are able to alleviate the negative affective states associated with acute nicotine withdrawal (Patterson et al. 2009b). So, the finding of partially divergent effects of nicotine and varenicline on reward salience has been proposed as the reason for the superior efficacy of varenicline for smoking cessation over nicotine replacement treatment (Fedota et al. 2015; Anthenelli et al. 2016).

Evaluating more closely the reviewed results as they pertain to cravings, according to the results of the Franklin et al. study, varenicline treatment was correlated with diminished cue-induced ventral striatum and mOFC activation and this was concomitant with reduced cravings induced by smoking-related cues (Franklin et al. 2011). This is in line with the bupropion-induced decreased cue-induced activation in the left ventral striatum and right mOFC (Culbertson et al. 2011). In addition, in the same study a decrease in cravings was associated with reduced activation of the mOFC (as well as the left ACC) irrespectively of the treatment received (placebo vs bupropion) (Culbertson et al. 2011). Similarly, deactivations of the OFC and the ventral striatum have been reported with the administration of nicotine with an electronic cigarette in non-deprived smokers following smoking-related visual cues (Wall et al. 2017). In addition, abstinence-induced cravings have being correlated with activation of the OFC (Wang et al. 2007) and an increase in cravings has been associated with increased connectivity of the enhanced medial PFC network which includes the OFC in non-deprived smokers at rest (Janes et al. 2014). Also, the activation of the lOFC at rest during varenicline treatment predicted a diminished cue-induced response of the mOFC (Franklin et al. 2011). Based on these results, it could be hypothesized that the effect of varenicline to the lOFC is to reduce the cue-induced activity of the mOFC leading to decreased cue-induced cravings (Franklin et al. 2011). Interestingly, in a more recent study, a larger grey matter volume of the left OFC was associated with a positive treatment outcome with varenicline, implicating again this area in the mechanism of action of varenicline (Qian et al. 2019). Generally, cravings represent a core symptom of addiction, and appear to play a pivotal role in triggering relapse thus maintaining substance use (Ferguson and Shiffman 2009; Brewer et al. 2013). The clinical significance of cravings is illustrated by the fact that the intensity of cravings during the initial stages of quitting is a predictor of relapse and by the fact that the intensity of cravings real-time is associated with imminent relapse (Shiffman et al. 1996, 1997). Recognizing their diagnostic and clinical importance, the American Psychiatric Association added cravings as one of the diagnostic criteria of substance use disorders in the latest edition of the DSM diagnostic manual (American Psychiatric Association 2013). Smoking-related cues have been shown to induce activation in key components of brain function that determine behavior, including the PFC (regulating attention), the amygdala (involved in emotion regulation and processing), the ventral tegmental area (involved in reward processing) and the striatum (involved in motivation)(Brody et al. 2002; Due et al. 2002; David et al. 2005; Lee et al. 2005; Wang et al. 2007; Culbertson et al. 2011).

In contrast, cravings per se as mentioned earlier have been associated with OFC activation (Brody et al. 2002). The OFC, occupying the most ventral part of the PFC, appears to play an important role in associative learning, incorporating information regarding the reward value of stimuli and participating in decision-making. In fact, the different anatomical parts (medial and lateral) appear to have distinct functions with the lateral aspect being more active when the reward value is relatively low, and the medial part being more active when the reward value is high (Rolls 2004; Elliott et al. 2010; Franklin et al. 2011; Pelletier and Fellows 2021). Reduced thickness of the mOFC has been reported in smokers and has been correlated inversely with the amount of cigarettes smoked per day possibly indicating reward system dysfunction, impulse control and decision-making aberrations (Kühn et al. 2010). Impairments of the OFC may result in enhanced stress reactivity, inability to contain emotional moods (Bechara et al. 2000), increased intention to seek drugs (Rolls 2000), and a poorer ability to control substance-related cue-induced behaviours (Volkow and Fowler 2000). As such and in light of the important role of the OFC in reward processing and decision making in the context of craving (Elliott et al. 2010; Plassmann et al. 2010), it is perhaps not surprising that several studies have identified varenicline-induced changes at the level of the OFC which may at least in part underline the anticraving properties of this medication as discussed earlier (Franklin et al. 2011). These findings may have implications towards the development of very promising and cost-effective future treatments based on the paradigm of audiovisual smoking-cue reactivity, with OFC and other aforementioned anatomical regions representing prime research targets. A deeper understanding of the neurobiology of cravings will hopefully lead to treatments that more adequately address this important part of the addictive process.

### Impact of varenicline on attention and working memory

Attention and working memory appear to be impaired during early abstinence and these deficits improve with varenicline treatment (or nicotine) especially in highly dependent smokers with tasks of higher difficulty (Atzori et al. 2008; Myers et al. 2008; Loughead et al. 2010). Alleviation of nicotine withdrawal-induced deficits has been observed with smoking cessation treatment with nicotine replacement (Lawrence et al. 2002; Hahn et al. 2007, 2009; Sweet et al. 2010) or bupropion (Perkins et al. 2013). This is also the case for varenicline, even though it has been reported that its effects on working memory and attention may be somewhat less consistent (Sofuoglu et al. 2009; Ashare and McKee 2012). Several brain areas were highlighted in the aforementioned study by Loughead et al. on varenicline’s effects with an emphasis on the increased activity of the dorsolateral PFC, the medial frontal cortex and the dorsal ACC during nicotine withdrawal in comparison to placebo (Loughead et al. 2010). Increased dopamine release induced by varenicline in the PFC via the stimulation of the α4β2 receptors and the α7 nAChRs has been suggested from a pharmacological standpoint to explain the aforementioned findings based on animal studies (Livingstone et al. 2009; Loughead et al. 2010). Comparing with the literature of similar studies on smokers not receiving treatment and utilizing similar tasks examining inhibitory control, increased activation of the bilateral inferior frontal gyrus during satiety and even more so during withdrawal has previously been shown (Chaarani et al. 2018). According to the literature on nicotine replacement treatment and its effects on brain function, during visual sustained attention testing, the patietal cortex, the occipital cortex, the thalamus and the caudate have been shown to exhibit increased activation whereas the parahippocampal gyrus and insula exhibit decreased activation (Lawrence et al. 2002). During a visuospatial attention task, nicotine replacement treatment has been shown to induce decreased activity in the ACC -in contrast to the aforementioned results on varenicline (Loughead et al. 2010)- as well as the posterior cingulate, the left angular gyrus, the left middle frontal gyrus and the bilateral cuneus (Hahn et al. 2007). During selective and divided attention tasks, decreased activity of the medial frontal gyrus, the rostral ACC, the left middle temporal gyrus and the parahippocampal gyrus has been observed with nicotine administration in smokers (Hahn et al. 2009). During resting fMRI, areas including the left dorso-medial PFC -please note that the dorsolateral PFC was implicated in the Loughead study above (Loughead et al. 2010)-, the left thalamus, the left OFC have been correlated with a reduction in the nicotine withdrawal-related symptoms with nicotine administration following abstinence (Cole et al. 2010). Hence, it appears that there is a degree of convergence in that NRT or varenicline can suppress the function of the ACC and the PCC under specific testing conditions, whereas varenicline can stimulate the ACC under different conditions suggesting a task- or condition-dependent pattern of action for alleviating the nicotine-withdrawal-related cognitive deficits. However, it should be noted that a full comparison between the results of different studies is limited to some extent by the use of different methodology, including the administration of different tasks among different studies.

In addition, a separate study illustrated that the activation in distributed areas involved in alertness, learning and memory (the right insular cortex, the left anterior thalamus, the bilateral middle cingulate, the posterior cingulate gyrus) while resisting the urge to smoke prior to a quit attempt with varenicline correlates with a successful outcome (Hartwell et al. 2013) indicating that varenicline may be particularly effective in the subset of smokers showing an improvement in cognitive deficits. Of note, reduced baseline FC between the left dorsomedial thalamus and the left cerebellum has been associated with treatment failure with varenicline (Qian et al. 2019), hence illustrating the potential of certain connectivity patterns being used as predictive biomarkers of treatment success. Interestingly, in a separate non varenicline related study, relapse was predicted by decreased left dorsolateral PFC and increased PCC BOLD signal change when comparing abstinence to smoking satiety during a working memory visual task (Loughead et al. 2015). Basically, nicotine appears to induce the deactivationof areas of the default network prior to the onset of an attentional task and to enhance the alerting effects of external stimuli during early abstinence (Hahn et al. 2007).

In summary, the aforementioned studies suggest that cognitive deficits especially during nicotine withdrawal and also executive dysfunction appear to be correlated with relapse (Moss et al. 2009; Loughead et al. 2010, 2015; Patterson et al. 2010) therefore also representing an important target for future treatment approaches with the hope that if they could be alleviated by a therapeutic agent, the chances of relapse could also be decreased. Important areas of the DMN including the PCC and the precuneus appear to be suppressed by varenicline under certain conditions indicating that the deactivation of the DMN by varenicline is also involved in its cognitive-enhancing properties during nicotine withdrawal. The importance of the suppression of OFC activation and the stimulation of the PFC by varenicline in possibly decreasing automatic drug-taking behavior has been previously emphasized. In addition, one could foresee a potential application of this research by which brain activity patterns derived from baseline testing of an attentional task with fMRI be used as a predictive biomarker of varenicline’s effectiveness in smokers interested in pursuing smoking cessation treatment, thus improving treatment outcomes.

### Impact of varenicline on emotional regulation

In terms of emotional regulation, based on the results of the current review, varenicline decreases cue-induced activation of the amygdala but not in a task-related manner as would have been expected during a face emotional reactivity task. At the same time, varenicline did appear to activate areas of the face processing system (occipital cortex, middle temporal gyrus) (Loughead et al. 2013). No major changes in amygdala reactivity were detected in a separate study with varenicline (Sutherland et al. 2013b). The discrepancy suggests that the effects of varenicline may not be uniform among all smokers and may depend on unknown underlying variables. In addition, increased resting-state interactions demonstrated between the amygdala and insula, as well as the insula and the default network during early abstinence were not alleviated with varenicline and nicotine (Sutherland et al. 2013a). As mentioned earlier changes have been observed in the activity of the amygdala in response to smoking-related stimuli with nicotine replacement treatment as well (McClernon et al. 2007) suggesting that this may represent a common mechanism of action of these pharmacotherapies. In general, the aforementioned findings illustrate that the limbic structures (including the amygdala) but also the insula, that are important in emotional regulation, are stimulated in nicotine withdrawal possibly leading to increased craving and relapse within the early period of abstinence. Such limbic system activation may at least partly mediate negative affect and an increase in emotional reactivity present during withdrawal which constitutes a motivational trigger to relapse (Lerman et al. 2002; Baker et al. 2004; al’Absi et al. 2007). Nicotine and, contingent upon the task/condition, varenicline seem to be able to mitigate these changes (Kahler et al. 2002; Patterson et al. 2008, 2009a) suggesting a common mechanism of action presumably via the α4b2-Ach-M receptors.

### Impact of varenicline on the default-mode network

In terms of default-mode network abberations, decreased regional homogeneity in the PCC prior to treatment has been associated with treatment failure with varenicline (Wang et al. 2017). This indicates the possibility of involvement of a key part of the default-mode network (the PCC) (Buckner et al. 2008) in the relapse process. Interestingly, varenicline treatment has been correlated with decreased activation of the ACC, the mOFC, the PCC during an executive function-related task indicating effects on the default mode network as presented above (Wheelock et al. 2014). Decreased resting-state FC between the dorsal ACC and the ventral striatum has been associated inversely with increased severity of nicotine use disorder whereas nicotine administration does not alleviate these aberrations. This has been proposed as a possible reason for nicotine replacement treatment not being as effective for most smokers (Hong et al. 2009; Fedota and Stein 2015). No pertinent studies on resting-state FC or the default-mode network were identified in smokers receiving treatment with bupropion. In terms of its function, the default-mode network produces spontaneous fluctuations at rest, when an individual is not focused on external stimuli and is thought to be involved in self-referential processing and autobiographical memory retrieval (Buckner et al. 2008; Kim 2012). Dysfunction of the default-mode network has been shown to be present in multiple different substance use disorders and it has therefore been proposed as a promising target for addiction-related research for several different substances (Zhang and Volkow 2019). The aforementioned observations point towards the direction of the cingulate cortex as a point of convergence for the pathophysiology of nicotine use disorder, and suppresing areas of the DMN such as the PCC and the precuneus appear as promising strategies for future therapeutics.

### Commentary on methodology, limitations and future directions

Upon review of the aforementioned studies, one important observation is that there is significant heterogeneity in terms of the sample characteristics and the exact specifics of the protocol of each study. For instance, the sample size varied significantly with several studies having a moderate sample size with less than 25 smokers raising concerns about whether they possessed sufficient power to detect statistical differences (Loughead et al. 2010; Franklin et al. 2011; Hartwell et al. 2013; Sutherland et al. 2013a; b; Wheelock et al. 2014; Fedota et al. 2015; Lesage et al. 2017, 2020; Flannery et al. 2019). The subjects were treatment-seeking in some of the evaluated studies (Loughead et al. 2010, 2013; Hartwell et al. 2013; Wheelock et al. 2014; Shen et al. 2017; Wang et al. 2017, 2021) but not in others (Franklin et al. 2011; Sutherland et al. 2013a, b; Fedota et al. 2015; Lesage et al. 2017, 2020; Flannery et al. 2019). This is important in light of the fact that these groups appear to differ in several demographic and clinical characteristics, for instance in terms of rates of co-habitation with smokers, level of education, the rate of using complementary reinforcers to smoking (e.g. alcohol, coffee), and the degree of delay discounting (Rohde et al. 2004; Audrain-McGovern et al. 2009; Celma-Merola et al. 2018). The average daily use of cigarettes ranged from 16 cigarettes per day (Fedota et al. 2015) to 25 (Wheelock et al. 2014). The titration regimen of varenicline was the standard, recommended regimen by the manufacturer for all evaluated studies except for 2 studies where the titration regimen but not the target dose differed slightly (Franklin et al. 2011; Hartwell et al. 2013). However, the treatment duration with varenicline ranged considerably from 13 days (Loughead et al. 2010, 2013), 17 days (Sutherland et al. 2013a; Fedota et al. 2015; Flannery et al. 2019; Lesage et al. 2020), 5 weeks (Hartwell et al. 2013) to 12 weeks (Wheelock et al. 2014; Shen et al. 2017; Wang et al. 2017, 2020b). Abstinence was confirmed biochemically in several studies (Loughead et al. 2010, 2013; Hartwell et al. 2013; Wheelock et al. 2014; Shen et al. 2017; Wang et al. 2017). In others, the volunteers were allowed to or even instructed to smoke a last cigarette at different times prior to the imaging session (Franklin et al. 2011; Sutherland et al. 2013a; Wheelock et al. 2014; Fedota et al. 2015; Wang et al. 2017; Flannery et al. 2019; Qian et al. 2019). So, the length of abstinence prior to the scan differed considerably among different studies ranging from 35 min (Franklin et al. 2011), to at least 1 h (Wheelock et al. 2014), 12 h (Sutherland et al. 2013a; Fedota et al. 2015; Lesage et al. 2017, 2020; Flannery et al. 2019), 3 days (Loughead et al. 2010, 2013) or even several weeks (Shen et al. 2017; Wang et al. 2017; Qian et al. 2019). The reason for these vast differences in methodology was that in some studies the researchers were evaluating brain function during acute nicotine withdrawal, whereas in others during the satiated state. However, given that it is well-known that nicotine has transient and fluctuating acute effects on brain function ranging from intoxication to withdrawal on top of chronic effects (Wang et al. 2014; Mishra et al. 2015), the timing of the last cigarette, the nicotine stimulation status (acute withdrawal, subacute withdrawal versus satiety) and the smoking history constitute important factors to be considered carefully in the interpretation of each of these studies. In a few of the studies presented, the researchers were able to differentiate between state-like and trait-like characteristics by carefully testing smokers in both states (withdrawal versus satiety) and comparing them with non-smokers as well; in other words, they examined both the smoking trait (smokers versus non-smokers) and the nicotine state (withdrawal versus satiety), in the presence or absence of varenicline (Sutherland et al. 2013a; Lesage et al. 2017, 2020; Flannery et al. 2019). In contrast, in other studies this comparison was not an aspect under investigation based on the design of the study (Loughead et al. 2010, 2013; Franklin et al. 2011; Hartwell et al. 2013; Wheelock et al. 2014; Shen et al. 2017; Wang et al. 2017; Qian et al. 2019). In some studies, a double-blind, placebo-controlled, within-subject cross-over design was used (Loughead et al. 2010, 2013; Sutherland et al. 2013a; Fedota et al. 2015; Lesage et al. 2017, 2020; Flannery et al. 2019). In others, there was a double-blind, randomized, placebo-controlled, non cross-over design (Franklin et al. 2011). In some studies, concurrent treatment with nicotine replacement treatment was assessed providing additional insights, as the effects of nicotine withdrawal were controlled for, but also making the interpretation of the results more complex (Sutherland et al. 2013a; Fedota et al. 2015; Flannery et al. 2019; Lesage et al. 2020). These are important considerations, especially when attempting to disentangle the effects of chronic smoking, quitting smoking and its accompanying nicotine withdrawal syndrome from direct medication effects. In other studies, the design was open-label and not placebo controlled (Hartwell et al. 2013; Wheelock et al. 2014; Shen et al. 2017; Wang et al. 2017, 2021; Qian et al. 2019). Lastly, different tasks targeting different aspects of cognitive functions were used, including working memory (Loughead et al. 2010), emotional processing (Loughead et al. 2013; Sutherland et al. 2013b), reward-based learning (Fedota et al. 2015), cognitive control (Wheelock et al. 2014; Lesage et al. 2020), reward processing in response to smoking-related audiovisual (Franklin et al. 2011), visual cues (Hartwell et al. 2013) or performance feedback (Flannery et al. 2019). In some studies, the brain was assessed in its resting state principally (Sutherland et al. 2013a; Shen et al. 2017; Wang et al. 2017, 2021; Qian et al. 2019). In addition, in some of the studies all participants were exclusively male (Shen et al. 2017; Wang et al. 2017, 2021; Qian et al. 2019), whereas in all others both genders were included in a more balanced manner. In conclusion, the large degree of heterogeneity of the sample population in terms of multiple differing variables, but also of the study design, in the context of a limited set of available studies, illustrate not only the complex nature of assessing nicotine use disorder but also that further research is needed to confirm these results and ensure that they can be generalized to the target treatment population.

One interesting question posed as it pertains to nicotine use disorder (as well as addiction in general) is whether there are preexisting aberrations of brain function leading to substance use prior to exposure to the substance versus whether the substance use per se is responsible for the brain changes observed. In other words, distinguishing between the causes and the consequences of nicotine use disorder (McClernon 2009). Only large long-term prospective studies of healthy volunteers prior to exposure to smoking (and prior to treatment with varenicline) would be capable of potentially settling this question. To the best of our knowledge, long-term prospective studies evaluating these brain differences and the effects of varenicline do not currently exist. Differences between smokers and non-smokers have been evaluated in several of the reviewed studies which compared the effects of varenicline among these two groups (Sutherland et al. 2013b; a; Fedota et al. 2015; Lesage et al. 2017, 2020; Flannery et al. 2019; Qian et al. 2019; Wang et al. 2020a). These however consist in exclusively cross-sectional studies which are only capable of detecting current differences between smokers and non-smokers. As such, they are not able to answer whether the differences preceded the onset of nicotine use disorder (and the treatment with varenicline) and whether they serve as predisposing factors for developing nicotine use disorder.

In an attempt to evaluate these findings from a more practical clinical perspective, the current review provides insights into the underlying neurological underpinnings of important clinical concepts as they pertain to varenicline’s mechanism of action and addiction treatment in general. For instance, the varenicline-induced diminished activation of the ventral striatum and the mOFC correlated with diminished cravings (Franklin et al. 2011), as well as the reduction of the salience of anticipated rewards, implicating the ACC (Fedota et al. 2015), both provide hints into the possible neurobiology underpinnings of cravings and incentive salience. It has to be noted, that it is not clear at this point what the correlation of cravings with diminished reactivity in certain parts of the reward system areas implies and the results of other studies have provided mixed results (Franklin et al. 2011). Nonetheless, some associations between behavioural outcomes and fMRI signal modulation effects of varenicline have been suggested as discussed above.

Current routine psychiatric practice as it pertains to smoking cessation treatment lacks objective testing that can assist the clinician in predicting treatment response. Development of such predictive biomarkers could represent the key to delivering care in a personalized manner, overcoming the delays and inefficiencies of current clinical care (Akil et al. 2010). Along these lines, the findings of brain activity patterns from all the reviewed studies regarding treatment outcome prediction based on baseline testing (prior to starting varenicline) are summarized in Table 9. A degree of convergent findings is noted for the thalamus and the insula with activation of these areas with smoking cues or increased resting-state FC with certain areas under the satiated state being predictive of successful treatment with varenicline. Interestingly, in one of the studies, increased thalamic volume was also associated with relapse (Wang et al. 2020a). Also, it should be mentioned that both of these areas possess abundant α4β2 nAChRs receptors (Picard et al. 2013) which may at least partly explain the effectiveness of varenicline, a partial α4β2 nAChR agonist, in modulating that activity. As mentioned earlier, varenicline can suppress the thalamus under nicotine withdrawal in a face emotion identification task (Loughead et al. 2013). It could be perceived that if the thalamus is hyperactive at baseline and this is predictive of treatment success, the ability of varenicline to suppress its activity under withdrawal may in part explain its mechanism of action. However, further research is needed to elucidate whether this is accurate especially given that not exactly the same areas were reported in these studies (left thalamus for the Wang et al. 2020a study vs. left anterior thalamus for the Hartwell et al. 2013 study vs. left dorsomedial thalamus for the Qian et al. 2019 study). Also, each of the studies was conducted under very different conditions: Loughead et al. used a face emotion identification task under withdrawal (Loughead et al. 2013), two studies evaluated the resting state under satiety (Qian et al. 2019; Wang et al. 2020a), and one study used smoking cues 2 h after smoking (Hartwell et al. 2013). In terms of the insula, a study demonstrated that increased activity in this region with cues at baseline 2 h after smoking was associated with varenicline treatment success (Hartwell et al. 2013). Interestingly, as discussed above, studies on the effects of varenicline during nicotine withdrawal have reported stimulation of this region (Lesage et al. 2017) while, at rest and under satiety, suppression of the activity of insula or of its FC with the default-mode network has been reported (Franklin et al. 2011; Sutherland et al. 2013a). As such, one would hypothesize that the high insular baseline activity is predictive of treatment success presumably due to the suppressive effect of varenicline in this region, at least under specific conditions during satiety. The nature of the association, if there is indeed one, between baseline insular activity and the stimulatory effect of the drug during nicotine withdrawal on this region is not clear. However, the condition-dependent opposing effect of the drug on this region is likely to reflect the partial agonist profile of varenicline with its dual pharmacological actions as discussed in the results section of this manuscript. Again, the studies were performed under very different conditions which render the interpretation of the combined findings very complex and full integration of the findings of potentially limited value. Methodological limitations do also exist especially in terms of the sample consisting exclusively of male and Chinese volunteers in many of the aforementioned studies (Qian et al. 2019; Wang et al. 2020a, b) and in terms of the open-label, non placebo-controlled protocol of all of the studies included in Table 9.

Brain patterns encountered during nicotine withdrawal and not alleviated by current treatments may explain their limitations. The relevant findings that pertain to varenicline are therefore summarized in Table 10. The decreased activation in response to rewards of the ACC, the ventral and dorsal striatum during nicotine withdrawal that remains unaffected by varenicline treatment is highlighted. Interestingly, these changes are not alleviated by NRT either (Lesage et al. 2017; Flannery et al. 2019). They appear to represent trait-like facets of nicotine use disorder and are associated with aspects of withdrawal including negative affective states and anhedonia (characterized by decreased responses to natural rewards). They are important especially in light of the negative reinforcement that withdrawal induces which perpetuates the addiction cycle as explained earlier (Goldstein and Volkow 2002; Wise and Koob 2014; Batalla et al. 2017). As such, these brain aberrations may represent an unmet clinical need which remains untouched by current treatments and serve as fruitful targets for the development of future therapeutics that will complement or even replace varenicline and NRT.

**Table 10:**
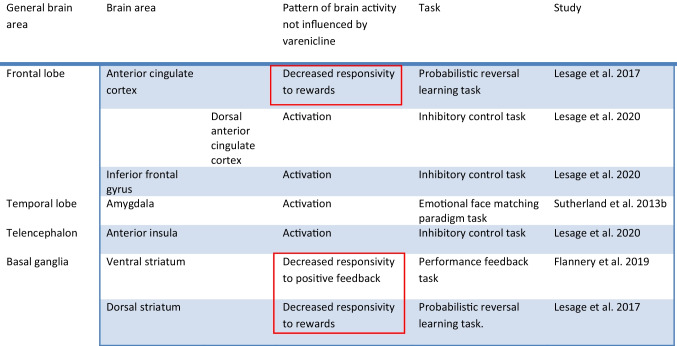
Brain activity patterns under nicotine withdrawal not affected by varenicline in fMRI studies

Based on the aforementioned and other related studiesit has been proposed that new diagnostic or screening tests could be developed in the future with the goal of constructing a personalized fMRI reactivity profile that could differentiate the population of smokers into subtypes. Separating subgroups could for instance provide valuable information about the nature of their vulnerability to relapse. Cue-vulnerable smokers are conceptualized as being more prone to relapse when exposed to smoking-related cues, whereas withdrawal-vulnerable smokers are more prone to relapse during the acute phase of the withdrawal. This is important especially in light of the finding that nicotine replacement treatment is more effective in reducing withdrawal-induced cravings but not as effective in reducing cue-induced cravings (Waters et al. 2004). Depending on the different subtype, a different, more individualized pharmacologic intervention could be chosen to address each patient’s relapse vulnerability profile (Franklin et al. 2011). In other words, future therapeutics have the potential to further enhance more traditional behavioural treatments. The classic relapse prevention strategy of avoidance of substance-related triggering stimuli (“Avoiding people, places and things”)(Melemis 2015) would be supplemented and reinforced with new pharmacological agents that induce diminished brain responses or a diminished reward value to the stimuli in the event of such an exposure to a triggering situation based on personalized neuroimaging data.

In terms of limitations of fMRI as a testing modality per se, it should be mentioned that one of the disadvantages of the method is that it requires the use of expensive equipment (Glover 2011). Another consideration is that task-based fMRI measurements at times suffer from low test–retest reliability reducing its value for longitudinal studies with this being less of a concern for resting-state fMRI measurements (Zuo and Xing 2014; Elliott et al. 2020). At the same time, one of its technical limitations in assessing brain function is its low temporal resolution (4–5 s) due to the fact that it relies on the indirect hemodynamic response (Kim et al. 1997; Lystad and Pollard 2009; Glover 2011). However, many of the physiologic processes involved in relapse including smoking cue reactivity appear to be happening very fast, namely within the range of milliseconds (Cui et al. 2013; Campanella et al. 2014). Thus, it is possible that some of the discrepancies presented in the current review could be explained by the low temporal resolution of fMRI testing. In light of this limitation, other testing modalities which offer better temporal resolution have been proposed as alternative or complementary techniques to fMRI including EEG (Michel et al. 2004; Lystad and Pollard 2009; Lopes da Silva 2013) or MEG tomography (Hämäläinen et al. 1993; Ilmoniemi and Näätänen 2001; Ioannides 2006; Lystad and Pollard 2009; Lopes da Silva 2013) in light of the fact that fMRI imaging as a method may not be ideal when it pertains to fast, rapidly evolving brain functions (Kim et al. 1997). Also, EEG-based testing, could prove useful especially taking into account that significant EEG changes have been observed with exposure to nicotine, nicotine withdrawal and abstinence (Gilbert et al. 1999, 2004; Domino 2003; Teneggi et al. 2004) and specific EEG changes have been associated with cravings in nicotine use disorder and other substance use disorders (Reid et al. 2003; Knyazev 2007, 2012; Littel et al. 2009; Wacker et al. 2009). In more detail, increased cravings have been associated with increases in the power of the delta band and the higher alpha band, and with reduced theta activity whereas beta power has been found to increase in smokers with smoking-related visual cues (Littel et al. 2009). Also, increased beta and theta EEG activity in smokers has been observed with smoking-related imagery (Knott et al. 2008) and low-theta EEG coherence with smoking cues has been reported as a predictor of cigarette cravings (Bu et al. 2019). Utilizing EEG-based testing could prove interesting because the EEG effects of varenicline have not been studied extensively with the exception of a few studies to the best of our knowledge (Rudnick et al. 2010; Versace et al. 2019).

In terms of limitations of the current study, it should be mentioned that it is possible that not all related studies were presented. The fact that only PubMed and Google Scholar were used as search databases may have potentially reduced the number of eligible studies. However, having allowed for the inclusion of studies from the references of any of the selected studies further reduced the impact of this methodological strategy to a minimum. The current team attempted to include all pertinent studies and to eliminate selection bias as much as possible by adhering to predefined criteria. The scope of the current review was somewhat limited in that only fMRI-related studies on smokers undergoing treatment with varenicline were included. This obviously limits the information to be presented regarding a multi-faceted illness such as nicotine use disorder. However, at the same time it allows for focusing on the most effective pharmacotherapy to date. In light of differing methodologies across research studies, no meta-analysis or calculation of effect sizes were conducted.

**Fig. 2:**
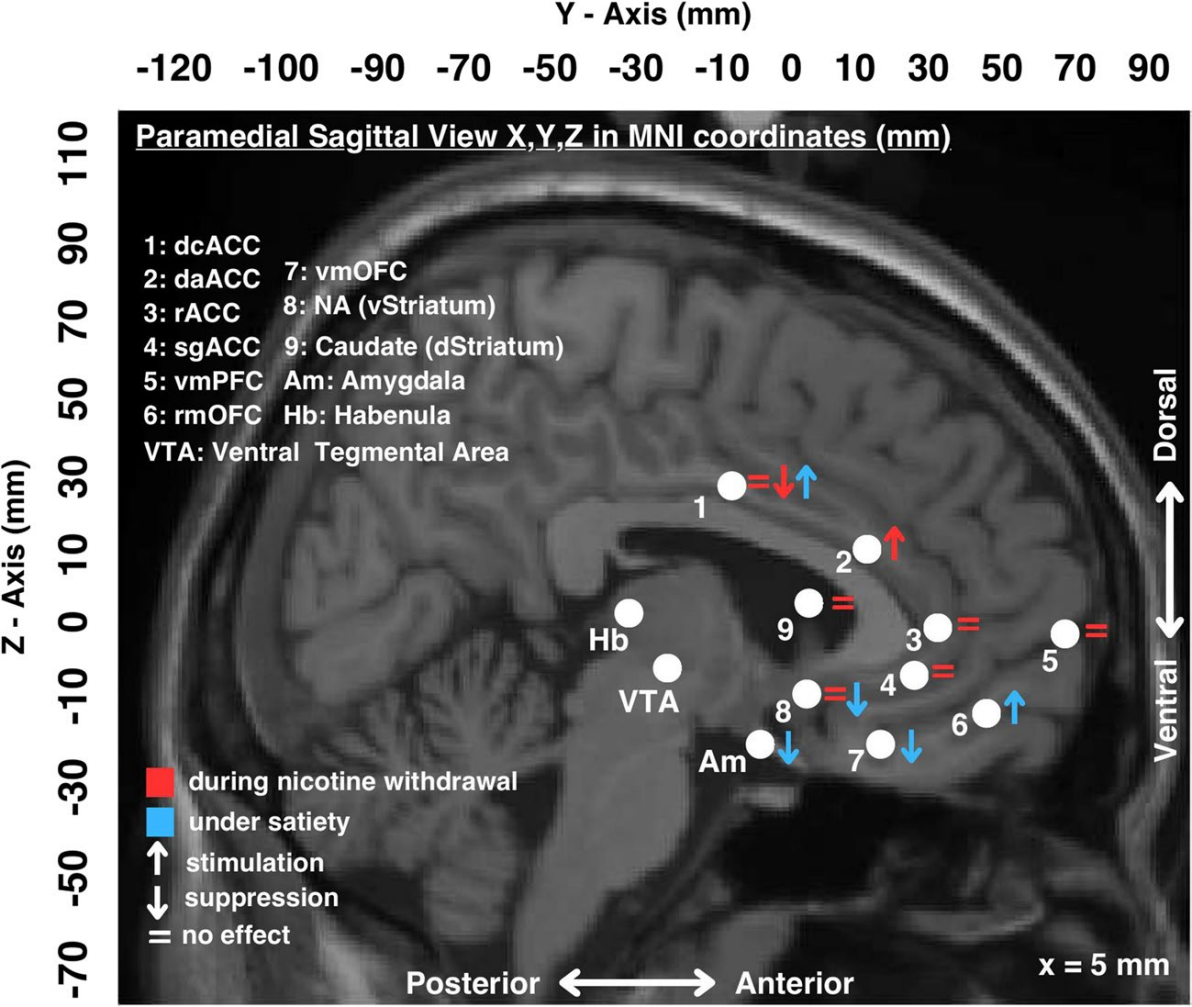
Key areas involved in nicotine use disorder and/or influenced by treatment with varenicline. The aforementioned areas are marked by a white symbol. During nicotine withdrawal and while performing specific tasks, decreased mesocorticolimbic activity (anterior cingulate cortex, striatum, anterior insula) and increased amygdala activity are alleviated by varenicline. However, other nicotine withdrawal-induced changes, including the decreased reward responsivity of the ventral striatum (vStriatum), the dorsal striatum (dStriatum) and the anterior cingulate cortex (ACC) are not influenced by varenicline while performing different tasks. During satiety, varenicline diminishes cue-induced activation of the vStriatum and the medial orbitofrontal cortex (mOFC), whereas at rest it stimulates the lateral orbitofrontal cortex (lOFC) and suppresses the right amygdala. The effects of varenicline are symbolized to the right of each corresponding area by an upwards or downwards arrow or the equals sign denoting stimulation, suppression of the area or no effect respectively; the red color denotes that the effect is taking place under nicotine withdrawal, whereas the blue color denotes satiety. Only areas adjacent to the midline are included in the current figure. Lateral areas and the different tasks performed are not included for clarity and brevity. A paramedial sagittal slice at the level of the habenula (Hb) of the colin27″ brain (in MNI152 space) (Holmes et al. 1998) was used; this plane is 5 mm lateral to the mid-sagittal plane. The Ventral Tegmental Area (VTA), is the only area on the mid sagittal plane. The four subdivisions of the ACC are represented by circles 1–4: The dorsal part (dACC), separated into its caudal (1: dcACC) and anterior part (2: daACC), the rostral ACC (3: rACC) and the subgenual ACC (4: sgACC). The ventral aspect of the medial prefrontal cortex (5: vmPFC) is depicted as area 5. The mOFC is separated into the rostral (6: rmOFC) and the more caudal and ventral (7: vmOFC) parts. These two mOFC areas extend to the lateral direction into the lOFC consisting in the rostral lateral OFC (rlOFC) and the narrow caudal ventral lateral Orbitofrontal cortex. The Nucleus Accumbens (8: NA), a part of the vStriatum, lies approximately 1 cm laterally and is shown at its orthographic projection. The Caudate Nucleus is on the medial side of the dStriatum. In the figure, the medial edge of the dStriatum is marked by a circle, about 1.5 cm dorsal to the NA (9: medial border of the ventral part of the Caudate Nucleus). The most lateral area displayed in the figure is the amygdalae seen at its orthographic projection approximately 2 cms away (Am: Amygdalae). The ACC, PFC, OFC and habenula are within 4 mm of the displayed slice, whereas the NA and caudate are more than 5 mm laterally

Several clues regarding the underlying pathophysiology of nicotine use disorder and underlying neurobiological phenomena that increase vulnerability to relapse have been identified in the aforementioned fMRI studies that further expand our understanding of this treatment-resistant illness. Several specific brain areas were highlighted as prime candidates for future studies in the current review, including the ventral and dorsal striatum, the ACC, the lOFC and mOFC, and the amygdala. Figure 2 serves as the visual epilogue of the current review with two objectives in mind. The first one is to provide a snapshot of the complexity of the effects of varenicline on brain function. Four of the reviewed articles were selected to be included based on their diligent methodological design and important findings. Three studies pertain to the effects of varenicline under nicotine withdrawal (Fedota et al. 2015; Lesage et al. 2017; Flannery et al. 2019) and one study pertains to the satiated state (Franklin et al. 2011). The figure demonstrates the complexity of the varenicline effects with clarity, showing for example differing effects in overlapping areas depending on the level of nicotine stimulation (withdrawal is depicted in red versus satiety which is depicted in blue color) as well as the nature of the task performed. The figure does not include more lateral areas which are important in nicotine use disorder, such as the insula and the dorsolateral PFC, for practical reasons. The second objective is to point out the relationship of our findings to a wider body of research beyond the narrow scope of the current review on varenicline-related neuroimaging studies. Therefore, areas close to the midline that were not highlighted prominently in the review, including the ventral tegmental area (VTA) and the habenula, are still being presented in the figure. The small size of the VTA and the habenula and the fact that their main action appears to consist in an early and short-lived (but critical) influence on other areas, particularly the ACC, might be the reason why they are not implicated as frequently in related functional neuroimaging studies in spite of their involvement in multiple neurotransmitter systems. Nevertheless, they appear to play an important role in nicotine use disorder (Kim and Picciotto 2023), reward processing and cognitive control (Baker et al. 2016), emotion and motivation (Ables et al. 2023) but also major depressive disorder (Browne et al. 2018). Their inclusion in Fig. 2 serves as a reminder of their importance, since they are key elements of the basic circuit controlling the midbrain dopaminergic system (Baker et al. 2016) which influences the ACC and frontal areas that are implicated in the studies reviewed. The link to broader studies and diagnostic modalities complements the findings of functional neuroimaging. The hope is that based on research on the aforementioned and other brain areas, the development of practical and economical biomarkers that can improve smoking cessation outcomes by predicting treatment response for individual patients would become feasible (Chen et al. 2014; Drysdale et al. 2017). In addition, neuroimaging testing could hopefully also be used as a predictor or surrogate marker of the effectiveness of candidate experimental medications thus accelerating the lengthy and tedious process of medication development research and ultimately leading to the development of much more effective and selective smoking cessation treatments than varenicline.

## References

[CR1] Ables JL, Park K, Ibañez-Tallon I (2023). Understanding the habenula: a major node in circuits regulating emotion and motivation. Pharmacol Res.

[CR2] Akil H, Brenner S, Kandel E, Kendler KS, King MC, Scolnick E (2010). Medicine. The future of psychiatric research: genomes and neural circuits. Science.

[CR3] al’Absi M, Carr SB, Bongard S (2007). Anger and psychobiological changes during smoking abstinence and in response to acute stress: prediction of smoking relapse. Int J Psychophysiol.

[CR4] American Cancer Society, A.C.S.I. (2015). The Tobacco Atlas.

[CR5] American Psychiatric Association (2013) Substance-related and addictive disorders*.* Washington, DC

[CR6] Anthenelli RM, Benowitz NL, West R, St Aubin L, McRae T, Lawrence D (2016). Neuropsychiatric safety and efficacy of varenicline, bupropion, and nicotine patch in smokers with and without psychiatric disorders (EAGLES): a double-blind, randomised, placebo-controlled clinical trial. Lancet.

[CR7] Ashare RL, McKee SA (2012). Effects of varenicline and bupropion on cognitive processes among nicotine-deprived smokers. Exp Clin Psychopharmacol.

[CR8] Atzori G, Lemmonds CA, Kotler ML, Durcan MJ, Boyle J (2008). Efficacy of a nicotine (4 mg)-containing lozenge on the cognitive impairment of nicotine withdrawal. J Clin Psychopharmacol.

[CR9] Audrain-McGovern J, Rodriguez D, Epstein LH, Rodgers K, Cuevas J, Wileyto EP (2009). Young adult smoking: what factors differentiate ex-smokers, smoking cessation treatment seekers and nontreatment seekers?. Addict Behav.

[CR10] Baker TB, Piper ME, McCarthy DE, Majeskie MR, Fiore MC (2004). Addiction motivation reformulated: an affective processing model of negative reinforcement. Psychol Rev.

[CR11] Baker PM, Jhou T, Li B, Matsumoto M, Mizumori SJ, Stephenson-Jones M (2016). The lateral habenula circuitry: reward processing and cognitive control. J Neurosci.

[CR12] Batalla A, Homberg JR, Lipina TV, Sescousse G, Luijten M, Ivanova SA (2017). The role of the habenula in the transition from reward to misery in substance use and mood disorders. Neurosci Biobehav Rev.

[CR13] Bechara A, Damasio H, Damasio AR (2000). Emotion, decision making and the orbitofrontal cortex. Cereb Cortex.

[CR14] Berridge KC (2017) Incentive motivation and incentive salience☆. In: Reference module in neuroscience and biobehavioral psychology. Elsevier. 10.1016/B978-0-12-809324-5.00342-4

[CR15] Brewer JA, Elwafi HM, Davis JH (2013). Craving to quit: psychological models and neurobiological mechanisms of mindfulness training as treatment for addictions. Psychol Addict Behav.

[CR16] Brody AL, Mandelkern MA, London ED, Childress AR, Lee GS, Bota RG (2002). Brain metabolic changes during cigarette craving. Arch Gen Psychiatry.

[CR17] Brody AL, Olmstead RE, London ED, Farahi J, Meyer JH, Grossman P (2004). Smoking-induced ventral striatum dopamine release. Am J Psychiatry.

[CR18] Browne CA, Hammack R, Lucki I (2018). Dysregulation of the lateral habenula in major depressive disorder. Front Synaptic Neurosci.

[CR19] Bu J, Ma R, Fan C, Sun S, Cheng Y, Piao Y (2019). Low-theta electroencephalography coherence predicts cigarette craving in nicotine addiction. Front Psychiatry.

[CR20] Buckner RL, Andrews-Hanna JR, Schacter DL (2008). The brain’s default network: anatomy, function, and relevance to disease. Ann N Y Acad Sci.

[CR21] Cahill K, Lindson-Hawley N, Thomas KH, Fanshawe TR, Lancaster T (2016). Nicotine receptor partial agonists for smoking cessation. Cochrane Database Syst Rev.

[CR22] Campanella S, Pogarell O, Boutros N (2014). Event-related potentials in substance use disorders: a narrative review based on articles from 1984 to 2012. Clin EEG Neurosci.

[CR23] Celma-Merola J, Abella-Pons F, Mata F, Pedra-Pagés G, Verdejo-Garcia A (2018). Self-changing behaviour in smoking cessation linked to trait and cognitive impulsivity. Addiction.

[CR24] Chaarani B, Spechler PA, Ivanciu A, Snowe M, Nickerson JP, Higgins ST (2018). Multimodal neuroimaging differences in nicotine abstinent smokers versus satiated smokers. Nicotine Tob Res.

[CR25] Chen LS, Bloom AJ, Baker TB, Smith SS, Piper ME, Martinez M (2014). Pharmacotherapy effects on smoking cessation vary with nicotine metabolism gene (CYP2A6). Addiction.

[CR26] Coe JW, Brooks PR, Vetelino MG, Wirtz MC, Arnold EP, Huang J (2005). Varenicline: an alpha4beta2 nicotinic receptor partial agonist for smoking cessation. J Med Chem.

[CR27] Cole DM, Beckmann CF, Long CJ, Matthews PM, Durcan MJ, Beaver JD (2010). Nicotine replacement in abstinent smokers improves cognitive withdrawal symptoms with modulation of resting brain network dynamics. Neuroimage.

[CR28] Community Preventive Services Task Force (2014) High-priority evidence gaps for clinical preventive services, 4th Annual Report to Congress. Reducing tobacco use and secondhand smoke exposure: smoke-free policies. https://www.thecommunityguide.org/pages/2015-annual-report-to-congress.html

[CR29] Community Preventive Services Task Force (2015) High-priority evidence gaps for clinical preventive services, 5th Annual Report to Congress. https://www.thecommunityguide.org/pages/2015-annual-report-to-congress.html 38011295

[CR30] Crunelle CL, Miller ML, de Bruin K, van den Brink W, Booij J (2009). Varenicline increases striatal dopamine D(2/3) receptor binding in rats. Addict Biol.

[CR31] Cui Y, Versace F, Engelmann JM, Minnix JA, Robinson JD, Lam CY (2013). Alpha oscillations in response to affective and cigarette-related stimuli in smokers. Nicotine Tob Res.

[CR32] Culbertson CS, Bramen J, Cohen MS, London ED, Olmstead RE, Gan JJ (2011). Effect of bupropion treatment on brain activation induced by cigarette-related cues in smokers. Arch Gen Psychiatry.

[CR33] David SP, Munafò MR, Johansen-Berg H, Smith SM, Rogers RD, Matthews PM (2005). Ventral striatum/nucleus accumbens activation to smoking-related pictorial cues in smokers and nonsmokers: a functional magnetic resonance imaging study. Biol Psychiatry.

[CR34] Domino EF (2003). Effects of tobacco smoking on electroencephalographic, auditory evoked and event related potentials. Brain Cogn.

[CR35] Drysdale AT, Grosenick L, Downar J, Dunlop K, Mansouri F, Meng Y (2017). Resting-state connectivity biomarkers define neurophysiological subtypes of depression. Nat Med.

[CR36] Due DL, Huettel SA, Hall WG, Rubin DC (2002). Activation in mesolimbic and visuospatial neural circuits elicited by smoking cues: evidence from functional magnetic resonance imaging. Am J Psychiatry.

[CR37] Elliott R, Agnew Z, Deakin JF (2010). Hedonic and informational functions of the human orbitofrontal cortex. Cereb Cortex.

[CR38] Elliott ML, Knodt AR, Ireland D, Morris ML, Poulton R, Ramrakha S (2020). What is the test-retest reliability of common task-functional mri measures? New empirical evidence and a meta-analysis. Psychol Sci.

[CR39] Esaki H, Deyama S, Izumi S, Katsura A, Nishikawa K, Nishitani N (2023). Varenicline enhances recognition memory via α7 nicotinic acetylcholine receptors in the medial prefrontal cortex in male mice. Neuropharmacology.

[CR40] Everitt BJ, Belin D, Economidou D, Pelloux Y, Dalley JW, Robbins TW (2008). Review. Neural mechanisms underlying the vulnerability to develop compulsive drug-seeking habits and addiction. Philos Trans R Soc Lond B Biol Sci.

[CR41] Fedota JR, Stein EA (2015). Resting-state functional connectivity and nicotine addiction: prospects for biomarker development. Ann N Y Acad Sci.

[CR42] Fedota JR, Sutherland MT, Salmeron BJ, Ross TJ, Hong LE, Stein EA (2015). Reward anticipation is differentially modulated by varenicline and nicotine in smokers. Neuropsychopharmacology.

[CR43] Ferguson SG, Shiffman S (2009). The relevance and treatment of cue-induced cravings in tobacco dependence. J Subst Abuse Treat.

[CR44] Fiore MC, Jaen CR (2008). A clinical blueprint to accelerate the elimination of tobacco use. JAMA.

[CR45] Flannery JS, Riedel MC, Poudel R, Laird AR, Ross TJ, Salmeron BJ (2019). Habenular and striatal activity during performance feedback are differentially linked with state-like and trait-like aspects of tobacco use disorder. Sci Adv.

[CR46] Franklin T, Wang Z, Suh JJ, Hazan R, Cruz J, Li Y (2011). Effects of varenicline on smoking cue–triggered neural and craving responses. Arch Gen Psychiatry.

[CR47] Garavan H, Pankiewicz J, Bloom A, Cho JK, Sperry L, Ross TJ (2000). Cue-induced cocaine craving: neuroanatomical specificity for drug users and drug stimuli. Am J Psychiatry.

[CR48] Gilbert DG, McClernon FJ, Rabinovich NE, Dibb WD, Plath LC, Hiyane S (1999). EEG, physiology, and task-related mood fail to resolve across 31 days of smoking abstinence: relations to depressive traits, nicotine exposure, and dependence. Exp Clin Psychopharmacol.

[CR49] Gilbert D, McClernon J, Rabinovich N, Sugai C, Plath L, Asgaard G (2004). Effects of quitting smoking on EEG activation and attention last for more than 31 days and are more severe with stress, dependence, DRD2 A1 allele, and depressive traits. Nicotine Tob Res.

[CR50] Giulietti F, Filipponi A, Rosettani G, Giordano P, Iacoacci C, Spannella F (2020). Pharmacological approach to smoking cessation: an updated review for daily clinical practice. High Blood Press Cardiovasc Prev.

[CR51] Glover GH (2011) Overview of functional magnetic resonance imaging. Neurosurg Clin N Am 22(2)**:**133–139, vii. 10.1016/j.nec.2010.11.001 10.1016/j.nec.2010.11.001 PMC3073717 21435566

[CR52] Goldstein RZ, Volkow ND (2002). Drug addiction and its underlying neurobiological basis: neuroimaging evidence for the involvement of the frontal cortex. Am J Psychiatry.

[CR53] Goldstein RZ, Volkow ND (2011). Dysfunction of the prefrontal cortex in addiction: neuroimaging findings and clinical implications. Nat Rev Neurosci.

[CR54] Gonzales D, Rennard SI, Nides M, Oncken C, Azoulay S, Billing CB (2006). Varenicline, an alpha4beta2 nicotinic acetylcholine receptor partial agonist, vs sustained-release bupropion and placebo for smoking cessation: a randomized controlled trial. JAMA.

[CR55] Hahn B, Ross TJ, Yang Y, Kim I, Huestis MA, Stein EA (2007). Nicotine enhances visuospatial attention by deactivating areas of the resting brain default network. J Neurosci.

[CR56] Hahn B, Ross TJ, Wolkenberg FA, Shakleya DM, Huestis MA, Stein EA (2009). Performance effects of nicotine during selective attention, divided attention, and simple stimulus detection: an fMRI study. Cereb Cortex.

[CR57] Hämäläinen M, Hari R, Ilmoniemi RJ, Knuutila J, Lounasmaa OV (1993). Magnetoencephalography–-theory, instrumentation, and applications to noninvasive studies of the working human brain. Rev Mod Phys.

[CR58] Hartwell KJ, Lematty T, McRae-Clark AL, Gray KM, George MS, Brady KT (2013). Resisting the urge to smoke and craving during a smoking quit attempt on varenicline: results from a pilot fMRI study. Am J Drug Alcohol Abuse.

[CR59] Hayashi T, Ko JH, Strafella AP, Dagher A (2013). Dorsolateral prefrontal and orbitofrontal cortex interactions during self-control of cigarette craving. Proc Natl Acad Sci U S A.

[CR60] Hickey C, van Zoest W (2012). Reward creates oculomotor salience. Curr Biol.

[CR61] Holmes CJ, Hoge R, Collins L, Woods R, Toga AW, Evans AC (1998). Enhancement of MR images using registration for signal averaging. J Comput Assist Tomogr.

[CR62] Hong LE, Gu H, Yang Y, Ross TJ, Salmeron BJ, Buchholz B (2009). Association of nicotine addiction and nicotine’s actions with separate cingulate cortex functional circuits. Arch Gen Psychiatry.

[CR63] Huang Z, Liang P, Jia X, Zhan S, Li N, Ding Y (2012). Abnormal amygdala connectivity in patients with primary insomnia: evidence from resting state fMRI. Eur J Radiol.

[CR64] Huang Y, Mohan A, De Ridder D, Sunaert S, Vanneste S (2018). The neural correlates of the unified percept of alcohol-related craving: a fMRI and EEG study. Sci Rep.

[CR65] Hughes JR (2013). An updated algorithm for choosing among smoking cessation treatments. J Subst Abuse Treat.

[CR66] Ilmoniemi RJ, Näätänen RJ, Smelser NJ, Baltes PB (2001). Magnetoencephalography. International encyclopedia of the social & behavioral sciences.

[CR67] Ioannides AA (2006). Magnetoencephalography as a research tool in neuroscience: state of the art. Neuroscientist.

[CR68] Janes AC, Frederick B, Richardt S, Burbridge C, Merlo-Pich E, Renshaw PF (2009). Brain fMRI reactivity to smoking-related images before and during extended smoking abstinence. Exp Clin Psychopharmacol.

[CR69] Janes AC, Farmer S, Frederick B, Nickerson LD, Lukas SE (2014). An increase in tobacco craving is associated with enhanced medial prefrontal cortex network coupling. PLoS ONE.

[CR70] Kahler CW, Brown RA, Ramsey SE, Niaura R, Abrams DB, Goldstein MG (2002). Negative mood, depressive symptoms, and major depression after smoking cessation treatment in smokers with a history of major depressive disorder. J Abnorm Psychol.

[CR71] Kaufman JN, Ross TJ, Stein EA, Garavan H (2003). Cingulate hypoactivity in cocaine users during a GO-NOGO task as revealed by event-related functional magnetic resonance imaging. J Neurosci.

[CR72] Kaur K, Kaushal S, Chopra SC (2009). Varenicline for smoking cessation: a review of the literature. Curr Ther Res Clin Exp.

[CR73] Kim H (2012). A dual-subsystem model of the brain’s default network: self-referential processing, memory retrieval processes, and autobiographical memory retrieval. Neuroimage.

[CR74] Kim K, Picciotto MR (2023). Nicotine addiction: more than just dopamine. Curr Opin Neurobiol.

[CR75] Kim SG, Richter W, Ugurbil K (1997). Limitations of temporal resolution in functional MRI. Magn Reson Med.

[CR76] Klumpp H, Hosseini B, Phan KL (2018). Self-reported sleep quality modulates amygdala resting-state functional connectivity in anxiety and depression. Front Psychiatry.

[CR77] Knott V, Cosgrove M, Villeneuve C, Fisher D, Millar A, McIntosh J (2008). EEG correlates of imagery-induced cigarette craving in male and female smokers. Addict Behav.

[CR78] Knyazev GG (2007). Motivation, emotion, and their inhibitory control mirrored in brain oscillations. Neurosci Biobehav Rev.

[CR79] Knyazev GG (2012). EEG delta oscillations as a correlate of basic homeostatic and motivational processes. Neurosci Biobehav Rev.

[CR80] Koob GF, Volkow ND (2010). Neurocircuitry of addiction. Neuropsychopharmacology.

[CR81] Koob GF, Volkow ND (2016). Neurobiology of addiction: a neurocircuitry analysis. Lancet Psychiatry.

[CR82] Kühn S, Schubert F, Gallinat J (2010). Reduced thickness of medial orbitofrontal cortex in smokers. Biol Psychiat.

[CR83] Lawrence NS, Ross TJ, Stein EA (2002). Cognitive mechanisms of nicotine on visual attention. Neuron.

[CR84] Lee JH, Lim Y, Wiederhold BK, Graham SJ (2005). A functional magnetic resonance imaging (FMRI) study of cue-induced smoking craving in virtual environments. Appl Psychophysiol Biofeedback.

[CR85] Lerman C, Audrain-McGovern J (2010). Reinforcing effects of smoking: more than a feeling. Biol Psychiatry.

[CR86] Lerman C, Roth D, Kaufmann V, Audrain J, Hawk L, Liu A (2002). Mediating mechanisms for the impact of bupropion in smoking cessation treatment. Drug Alcohol Depend.

[CR87] Lesage E, Aronson SE, Sutherland MT, Ross TJ, Salmeron BJ, Stein EA (2017). Neural signatures of cognitive flexibility and reward sensitivity following nicotinic receptor stimulation in dependent smokers: a randomized trial. JAMA Psychiat.

[CR88] Lesage E, Sutherland MT, Ross TJ, Salmeron BJ, Stein EA (2020). Nicotine dependence (trait) and acute nicotinic stimulation (state) modulate attention but not inhibitory control: converging fMRI evidence from go-nogo and flanker tasks. Neuropsychopharmacology.

[CR89] Li Q, Yang WC, Wang YR, Huang YF, Li W, Zhu J (2013). Abnormal function of the posterior cingulate cortex in heroin addicted users during resting-state and drug-cue stimulation task. Chin Med J (engl).

[CR90] Littel M, Franken IH, Van Strien JW (2009). Changes in the electroencephalographic spectrum in response to smoking cues in smokers and ex-smokers. Neuropsychobiology.

[CR91] Livingstone PD, Srinivasan J, Kew JN, Dawson LA, Gotti C, Moretti M (2009). alpha7 and non-alpha7 nicotinic acetylcholine receptors modulate dopamine release in vitro and in vivo in the rat prefrontal cortex. Eur J Neurosci.

[CR92] Lopes da Silva F (2013). EEG and MEG: relevance to neuroscience. Neuron.

[CR93] Loughead J, Ray R, Wileyto EP, Ruparel K, Sanborn P, Siegel S (2010). Effects of the alpha4beta2 partial agonist varenicline on brain activity and working memory in abstinent smokers. Biol Psychiatry.

[CR94] Loughead J, Ray R, Wileyto EP, Ruparel K, O’Donnell GP, Senecal N (2013). Brain activity and emotional processing in smokers treated with varenicline. Addict Biol.

[CR95] Loughead J, Wileyto EP, Ruparel K, Falcone M, Hopson R, Gur R (2015). Working memory-related neural activity predicts future smoking relapse. Neuropsychopharmacology.

[CR96] Luijten M, Littel M, Franken IH (2011). Deficits in inhibitory control in smokers during a Go/NoGo task: an investigation using event-related brain potentials. PLoS ONE.

[CR97] Lystad RP, Pollard H (2009). Functional neuroimaging: a brief overview and feasibility for use in chiropractic research. J Can Chiropr Assoc.

[CR98] Lyvers M, Carlopio C, Vicole Bothma H, Edwards MS (2014). Mood, mood regulation, and frontal systems functioning in current smokers, long-term abstinent ex-smokers, and never-smokers. J Psychoactive Drugs.

[CR99] Martín Ríos R, López-Torrecillas F, Martín Tamayo I (2021). Executive functions in tobacco use disorder: new challenges and opportunities. Front Psychiatry.

[CR100] McClernon FJ (2009). Neuroimaging of nicotine dependence: key findings and application to the study of smoking-mental illness comorbidity. J Dual Diagn.

[CR101] McClernon FJ, Hiott FB, Liu J, Salley AN, Behm FM, Rose JE (2007). Selectively reduced responses to smoking cues in amygdala following extinction-based smoking cessation: results of a preliminary functional magnetic resonance imaging study. Addict Biol.

[CR102] McClernon FJ, Kozink RV, Lutz AM, Rose JE (2009). 24-h smoking abstinence potentiates fMRI-BOLD activation to smoking cues in cerebral cortex and dorsal striatum. Psychopharmacology.

[CR103] Melemis SM (2015). Relapse prevention and the five rules of recovery. Yale J Biol Med.

[CR104] Menossi HS, Goudriaan AE, de Azevedo-Marques Périco C, Nicastri S, de Andrade AG, D’Elia G (2013). Neural bases of pharmacological treatment of nicotine dependence - insights from functional brain imaging: a systematic review. CNS Drugs.

[CR105] Michel CM, Murray MM, Lantz G, Gonzalez S, Spinelli L, Grave de Peralta R (2004). EEG source imaging. Clin Neurophysiol.

[CR106] Mishra A, Chaturvedi P, Datta S, Sinukumar S, Joshi P, Garg A (2015). Harmful effects of nicotine. Indian J Med Paediatr Oncol.

[CR107] Moss TG, Sacco KA, Allen TM, Weinberger AH, Vessicchio JC, George TP (2009). Prefrontal cognitive dysfunction is associated with tobacco dependence treatment failure in smokers with schizophrenia. Drug Alcohol Depend.

[CR108] Myers CS, Taylor RC, Moolchan ET, Heishman SJ (2008). Dose-related enhancement of mood and cognition in smokers administered nicotine nasal spray. Neuropsychopharmacology.

[CR109] National Center for Chronic Disease, P., Health Promotion Office on, S., and Health (2014) Reports of the surgeon general. In: The health consequences of smoking—50 years of progress: a report of the surgeon general. Centers for Disease Control and Prevention (US)), Atlanta

[CR110] Nides M, Glover ED, Reus VI, Christen AG, Make BJ, Billing CB (2008). Varenicline versus bupropion SR or placebo for smoking cessation: a pooled analysis. Am J Health Behav.

[CR111] Oliver JA, Evans DE, Addicott MA, Potts GF, Brandon TH, Drobes DJ (2017). Nicotine withdrawal induces neural deficits in reward processing. Nicotine Tob Res.

[CR112] Oncken C, Gonzales D, Nides M, Rennard S, Watsky E, Billing CB (2006). Efficacy and safety of the novel selective nicotinic acetylcholine receptor partial agonist, varenicline, for smoking cessation. Arch Intern Med.

[CR113] Pardo JV, Pardo PJ, Janer KW, Raichle ME (1990). The anterior cingulate cortex mediates processing selection in the stroop attentional conflict paradigm. Proc Natl Acad Sci U S A.

[CR114] Patterson F, Kerrin K, Wileyto EP, Lerman C (2008). Increase in anger symptoms after smoking cessation predicts relapse. Drug Alcohol Depend.

[CR115] Patterson F, Jepson C, Strasser AA, Loughead J, Perkins KA, Gur RC (2009). Varenicline improves mood and cognition during smoking abstinence. Biol Psychiatry.

[CR116] Patterson F, Jepson C, Strasser AA, Loughead J, Perkins KA, Gur RC (2009). Varenicline improves mood and cognition during smoking abstinence. Biol Psychiat.

[CR117] Patterson F, Jepson C, Loughead J, Perkins K, Strasser AA, Siegel S (2010). Working memory deficits predict short-term smoking resumption following brief abstinence. Drug Alcohol Depend.

[CR118] Paulus MP, Stein MB (2010). Interoception in anxiety and depression. Brain Struct Funct.

[CR119] Pelletier G, Fellows LK (2021). Viewing orbitofrontal cortex contributions to decision-making through the lens of object recognition. Behav Neurosci.

[CR120] Perkins KA, Karelitz JL, Jao NC, Gur RC, Lerman C (2013). Effects of bupropion on cognitive performance during initial tobacco abstinence. Drug Alcohol Depend.

[CR121] Pfizer Laboratories (2013) Medication guide: Chantix, https://www.pfizermedicalinformation.com/chantix/medguide

[CR122] Picard F, Sadaghiani S, Leroy C, Courvoisier DS, Maroy R, Bottlaender M (2013). High density of nicotinic receptors in the cingulo-insular network. Neuroimage.

[CR123] Plassmann H, O’Doherty JP, Rangel A (2010). Appetitive and aversive goal values are encoded in the medial orbitofrontal cortex at the time of decision making. J Neurosci.

[CR124] Prochaska JJ, Benowitz NL (2016). The past, present, and future of nicotine addiction therapy. Annu Rev Med.

[CR125] Qian W, Huang P, Shen Z, Wang C, Yang Y, Zhang M (2019). Brain gray matter volume and functional connectivity are associated with smoking cessation outcomes. Front Hum Neurosci.

[CR126] Reid MS, Prichep LS, Ciplet D, O’Leary S, Tom M, Howard B (2003). Quantitative electroencephalographic studies of cue-induced cocaine craving. Clin Electroencephalogr.

[CR127] Rohde P, Kahler CW, Lewinsohn PM, Brown RA (2004). Psychiatric disorders, familial factors, and cigarette smoking: III. Associations with cessation by young adulthood among daily smokers. Nicotine Tob Res.

[CR128] Rolls ET (2000). The orbitofrontal cortex and reward. Cereb Cortex.

[CR129] Rolls ET (2004). The functions of the orbitofrontal cortex. Brain Cogn.

[CR130] Rudnick ND, Strasser AA, Phillips JM, Jepson C, Patterson F, Frey JM (2010). Mouse model predicts effects of smoking and varenicline on event-related potentials in humans. Nicotine Tob Res.

[CR131] Shen Z, Huang P, Wang C, Qian W, Yang Y, Zhang M (2017). Increased network centrality as markers of relapse risk in nicotine-dependent individuals treated with varenicline. Prog Neuropsychopharmacol Biol Psychiatry.

[CR132] Shiffman SM, Jarvik ME (1976). Smoking withdrawal symptoms in two weeks of abstinence. Psychopharmacology.

[CR133] Shiffman S, Paty JA, Gnys M, Kassel JA, Hickcox M (1996). First lapses to smoking: within-subjects analysis of real-time reports. J Consult Clin Psychol.

[CR134] Shiffman S, Engberg JB, Paty JA, Perz WG, Gnys M, Kassel JD (1997). A day at a time: predicting smoking lapse from daily urge. J Abnorm Psychol.

[CR135] Sofuoglu M, Herman AI, Mooney M, Waters AJ (2009). Varenicline attenuates some of the subjective and physiological effects of intravenous nicotine in humans. Psychopharmacology.

[CR136] Suckling J, Nestor LJ (2017). The neurobiology of addiction: the perspective from magnetic resonance imaging present and future. Addiction.

[CR137] Sutherland MT, Carroll AJ, Salmeron BJ, Ross TJ, Hong LE, Stein EA (2013). Down-regulation of amygdala and insula functional circuits by varenicline and nicotine in abstinent cigarette smokers. Biol Psychiatry.

[CR138] Sutherland MT, Carroll AJ, Salmeron BJ, Ross TJ, Hong LE, Stein EA (2013). Individual differences in amygdala reactivity following nicotinic receptor stimulation in abstinent smokers. Neuroimage.

[CR139] Sweet LH, Mulligan RC, Finnerty CE, Jerskey BA, David SP, Cohen RA (2010). Effects of nicotine withdrawal on verbal working memory and associated brain response. Psychiatry Res.

[CR140] Teneggi V, Squassante L, Milleri S, Polo A, Lanteri P, Ziviani L (2004). EEG power spectra and auditory P300 during free smoking and enforced smoking abstinence. Pharmacol Biochem Behav.

[CR141] Tricomi E, Balleine BW, O’Doherty JP (2009). A specific role for posterior dorsolateral striatum in human habit learning. Eur J Neurosci.

[CR142] Versace F, Stevens EM, Robinson JD, Cui Y, Deweese MM, Engelmann JM (2019). Brain responses to cigarette-related and emotional images in smokers during smoking cessation: no effect of varenicline or bupropion on the late positive potential. Nicotine Tob Res.

[CR143] Volkow ND, Fowler JS (2000). Addiction, a disease of compulsion and drive: involvement of the orbitofrontal cortex. Cereb Cortex.

[CR144] Volkow ND, Wang GJ, Fowler JS, Tomasi D, Telang F, Baler R (2010). Addiction: decreased reward sensitivity and increased expectation sensitivity conspire to overwhelm the brain’s control circuit. BioEssays.

[CR145] Wacker J, Dillon DG, Pizzagalli DA (2009). The role of the nucleus accumbens and rostral anterior cingulate cortex in anhedonia: integration of resting EEG, fMRI, and volumetric techniques. Neuroimage.

[CR146] Wall MB, Mentink A, Lyons G, Kowalczyk OS, Demetriou L, Newbould RD (2017). Investigating the neural correlates of smoking: feasibility and results of combining electronic cigarettes with fMRI. Sci Rep.

[CR147] Wang Z, Faith M, Patterson F, Tang K, Kerrin K, Wileyto EP (2007). Neural substrates of abstinence-induced cigarette cravings in chronic smokers. J Neurosci.

[CR148] Wang K, Yang J, Zhang S, Wei D, Hao X, Tu S (2014). The neural mechanisms underlying the acute effect of cigarette smoking on chronic smokers. PLoS ONE.

[CR149] Wang C, Shen Z, Huang P, Qian W, Yu X, Sun J (2017). Altered spontaneous activity of posterior cingulate cortex and superior temporal gyrus are associated with a smoking cessation treatment outcome using varenicline revealed by regional homogeneity. Brain Imaging Behav.

[CR150] Wang C et al (2020a) Increased thalamic volume and decreased thalamo-precuneus functional connectivity are associated with smoking relapse. Neuroimage Clin 28:102451 10.1016/j.nicl.2020.102451 PMC7548987 33022581

[CR151] Wang C (2020). Increased interregional functional connectivity of anterior insula is associated with improved smoking cessation outcome. Brain Imaging Behav.

[CR152] Wang C, Huang P, Shen Z, Qian W, Wang S, Jiaerken Y (2021). Increased striatal functional connectivity is associated with improved smoking cessation outcomes: a preliminary study. Addict Biol.

[CR153] Waters AJ, Shiffman S, Sayette MA, Paty JA, Gwaltney CJ, Balabanis MH (2004). Cue-provoked craving and nicotine replacement therapy in smoking cessation. J Consult Clin Psychol.

[CR154] Weinstein A, Greif J, Yemini Z, Lerman H, Weizman A, Even-Sapir E (2010). Attenuation of cue-induced smoking urges and brain reward activity in smokers treated successfully with bupropion. J Psychopharmacol.

[CR155] Wheelock MD, Reid MA, To H, White DM, Cropsey KL, Lahti AC (2014). Open label smoking cessation with varenicline is associated with decreased glutamate levels and functional changes in anterior cingulate cortex: preliminary findings. Front Pharmacol.

[CR156] Wise RA, Koob GF (2014). The development and maintenance of drug addiction. Neuropsychopharmacology.

[CR157] Zhang R, Volkow ND (2019). Brain default-mode network dysfunction in addiction. Neuroimage.

[CR158] Zhu S-H, Melcer T, Sun J, Rosbrook B, Pierce JP (2000). Smoking cessation with and without assistance: a population-based analysis. Am J Prev Med.

[CR159] Zilcha-Mano S, Solomonov N, Posner JE, Roose SP, Rutherford BR (2022) Proof of concept of the contribution of the interaction between trait-like and state-like effects in identifying individual-specific mechanisms of action in biological psychiatry. J Pers Med 12(8). 10.3390/jpm12081197 10.3390/jpm12081197 PMC9332605 35893291

[CR160] Zuo XN, Xing XX (2014). Test-retest reliabilities of resting-state FMRI measurements in human brain functional connectomics: a systems neuroscience perspective. Neurosci Biobehav Rev.

